# Genome-wide identification and comparative evolutionary analysis of the Dof transcription factor family in physic nut and castor bean

**DOI:** 10.7717/peerj.6354

**Published:** 2019-02-05

**Authors:** Zhi Zou, Xicai Zhang

**Affiliations:** 1 Key Laboratory of Biology and Genetic Resources of Tropical Crops, Ministry of Agriculture and Rural Affairs, Institute of Tropical Biosciences and Biotechnology, Chinese Academy of Tropical Agricultural Sciences, Haikou, Hainan, P. R. China; 2 Danzhou Investigation & Experiment Station of Tropical Crops, Ministry of Agriculture and Rural Affairs, Rubber Research Institute, Chinese Academy of Tropical Agricultural Sciences, Haikou, Hainan, P. R. China

**Keywords:** *Jatropha curcas*, *Ricinus communis*, Dof gene family, Phylogenetic analysis, Orthologous group, Synteny analysis, Transcriptional profiling

## Abstract

DNA-binding with one finger (Dof) proteins comprise a plant-specific transcription factor family involved in plant growth, development and stress responses. This study presents a genome-wide comparison of Dof family genes in physic nut (*Jatropha curcas*) and castor bean (*Ricinus communis*), two Euphorbiaceae plants that have not experienced any recent whole-genome duplication. A total of 25 or 24 Dof genes were identified from physic nut and castor genomes, respectively, where JcDof genes are distributed across nine out of 11 chromosomes. Phylogenetic analysis assigned these genes into nine groups representing four subfamilies, and 24 orthologous groups were also proposed based on comparison of physic nut, castor, *Arabidopsis* and rice Dofs. Conserved microsynteny was observed between physic nut and castor Dof-coding scaffolds, which allowed anchoring of 23 RcDof genes to nine physic nut chromosomes. In contrast to how no recent duplicate was present in castor, two tandem duplications and one gene loss were found in the Dof gene family of physic nut. Global transcriptome profiling revealed diverse patterns of Jc/RcDof genes over various tissues, and key Dof genes involved in flower development and stress response were also identified in physic nut. These findings provide valuable information for further studies of Dof genes in physic nut and castor.

## Introduction

DNA-binding with one finger (Dof) proteins comprise a plant-specific transcription factor family, which is defined by the presence of the highly conserved Dof domain at the N-terminus (Yanagisawa, 2002). The Dof domain, which includes approximately 52 amino acids with a C_2_C_2_ zinc finger structure, mediates DNA binding as well as protein–protein interactions (Yanagisawa & Schmidt, 1999; Rueda-Romero et al., 2012). By contrast, the C-terminal region of Dof proteins, which usually contains a transcriptional activation domain, is highly variable (Yanagisawa, 2002; Krebs, Mueller-Roeber & Ruzicic, 2010). Genome-wide surveys showed that the Dof gene family is present as a single member in most algae, however, the family has been highly expanded and diversified in terrestrial plants (Moreno-Risueno et al., 2007; Gupta et al., 2015). For example, there are 25 Dof genes present in grapevine (*Vitis vinifera*) (da Silva et al., 2016), 30 in rice (*Oryza sativa*) (Lijavetzky, Carbonero & Vicente-Carbajosa, 2003), 33 in pepper (*Capsicum annuum*) (Wu et al., 2016), 36 members in *Arabidopsis thaliana* (Yanagisawa, 2002), 36 in cucumber (*Cucumis sativus*) (Wen et al., 2016), 41 in poplar (*Populus trichocarpa*) (Yang, Tuskan & Cheng, 2006), 45 in cassava (*Manihot esculenta*) (Zou, Zhu & Zhang, 2019b), and 76 in Chinese cabbage (*Brassica rapa*) (Ma et al., 2015). Based on phylogenetic analysis of Dof domains, Dof proteins in model plants were classed into four subfamilies and several groups (Lijavetzky, Carbonero & Vicente-Carbajosa, 2003). Functional analysis performed in several species also revealed diverse roles of Dof proteins in various biological processes, for example, light-mediated regulation, vascular system development, flowering control, flower abscission, pollen development, endosperm development, seed development and germination, carbon and nitrogen metabolism, hormone, and stress responses (Yanagisawa, 2002; Noguero et al., 2013; Gupta et al., 2015; Ma et al., 2015).

Physic nut (*Jatropha curcas* L., 2*n* = 22) and castor bean (*Ricinus communis* L., 2*n* = 20) are two economically important species that belong to the Euphorbiaceae family (Zou et al., 2016a, 2018). Based on analysis of single-copy gene families, physic nut and castor were estimated to diverge from a common ancestor at approximately 49.4 Mya (Wu et al., 2015). Also, comparative genomics analyses revealed that these two species didn’t experience any recent whole-genome duplication (WGD) after the ancient so-called γ event shared by all core eudicots, in contrast to two additional WGDs occurred in the model dicot *Arabidopsis* (Bowers et al., 2003; Chan et al., 2010; Jiao et al., 2012; Wu et al., 2015). From this perspective, analysis of Dof family genes in physic nut and castor may improve our knowledge on the evolution of this special gene family. Thus far, two Dof genes (i.e., *JcDof1* and *JcDof3*) have been characterized in physic nut (Yang et al., 2010, 2011). These two genes were isolated from seedlings by using yeast one-hybrid, which exhibit high sequence similarity to several circadian-regulated genes, for example, *CDFs* (cycling Dof factors) in *Arabidopsis* and *Rdds* (rice Dof daily fluctuations) (Imaizumi et al., 2005; Fornara et al., 2009; Iwamoto, Higo & Takano, 2009). Indeed, their expression was characterized by a circadian-clock oscillation under long day, short day and continuous light conditions, in contrast to arrhythmic expression at basal level in etiolated cotyledons under continuous dark conditions (Yang et al., 2010, 2011). Moreover, yeast two-hybrid screening indicated that JcDof1 and JcDof3 proteins can also interact with several *F*-box proteins that were shown to regulate photoperiodic flowering (Yang et al., 2011). Very recently, 22 more Dof genes were also identified from the physic nut genome, and their expression profiles upon various abiotic stresses and hormonal treatments were investigated based on publicly available RNA sequencing (RNA-seq) data (Wang et al., 2018). In castor, a genome-wide survey resulted in 21 RcDof genes, and semi-quantitative RT-PCR analysis revealed that 18 RcDof genes are responsive to abscisic acid (ABA) and/or gibberellic acid (Jin, Chandrasekaran & Liu, 2014). However, the homology search performed in these two studies was based on proteomes derived from automatic genome annotation (Jin, Chandrasekaran & Liu, 2014; Wang et al., 2018), leading to omission of several family members as well as incorrect gene expression annotation. Moreover, species-specific evolution of the family in these two plants is yet to be resolved.

In this study, we would like to present a genome-wide identification of the complete set of Dof family genes in physic nut and castor by taking advantage of the available genome as well as various transcriptome datasets. After an expert revision of their gene structures, sequence characteristics, chromosome locations, evolutionary relationships, and expression profiles were further investigated and comprehensively compared. These findings provide valuable information for further studies of Dof genes in physic nut and castor.

## Materials and Methods

### Datasets and sequence retrieval

As shown in Table S1, *Arabidopsis* and rice Dof genes described before were retrieved from The Arabidopsis Information Resource (TAIR10, http://www.arabidopsis.org/) and Rice Genome Annotation Project (RGAP7, http://rice.plantbiology.msu.edu/), respectively. Castor genomic sequences were accessed from Phytozome v12 (https://phytozome.jgi.doe.gov/pz/portal.html). The physic nut genome and other data such as nucleotides, Sanger expressed sequence tags (ESTs) and RNA-seq reads were obtained from NCBI (http://www.ncbi.nlm.nih.gov/, last accessed January 2018).

### Identification and manual curation of Dof family genes

The hidden Markov model (HMM)-based profile of the Dof domain (PF02701) was obtained from the Pfam database (http://pfam.sanger.ac.uk/), and was used as a query to perform the HMMER search (Version 3.0, http://hmmer.janelia.org/). The resulted protein sequences were further adopted for the TBLASTN search (Altschul et al., 1997), and the positive genomic sequences with an *E*-value of less than 1*e−*5 were predicted as described before (Zou et al., 2015a). Predicted gene models were further validated with available cDNAs, ESTs, and RNA-seq reads. The presence of the Dof domain was confirmed using SMART (http://smart.embl-heidelberg.de/). Homology search for nucleotides or ESTs and expression annotation using RNA-seq data were performed as described before (Zou et al., 2015b).

### Sequence alignment and phylogenetic analysis

Multiple sequence alignments were performed using MUSCLE (Edgar, 2004) and phylogenetic trees were constructed using MEGA (version 6.0) (Tamura et al., 2013) with the maximum likelihood method (bootstrap: 1,000 replicates). The alignment of Dof domains was displayed using Boxshade (http://www.ch.embnet.org/software/BOX_form.html), and gene structures were displayed using GSDS (Hu et al., 2015) via aligning the cDNA to the gene sequence. For synteny analysis, the all-to-all BLASTP method was used to identify duplicate pairs as previously described (Zou et al., 2018), and syntenic blocks were inferred using MCScanX (Wang et al., 2012). Considering the relatively fragmented status of the current castor genome assembly, MicroSyn (Cai et al., 2011) was also used to detect microsynteny. Best reciprocal hit (BRH)-based BLAST analysis described before (Zou et al., 2017a; Zou, Xie & Yang, 2017b) was also used to define orthologs across different species, where the *E*-value was set to 1*e*–10. Systematic names were assigned based on the phylogenetic analysis for castor, or chromosome distribution for physic nut.

### Protein properties and conserved motif analysis

Theoretical molecular weight (MW), isoelectric point (*p*I), and grand average of hydropathicity (GRAVY) were determined using ProtParam (http://web.expasy.org/protparam/). Protein subcellular localization was predicted using CELLO (http://cello.life.nctu.edu.tw/). Conserved motifs in different Dof proteins were analyzed using MEME (http://meme-suite.org/tools/meme), and the optimized parameters were: any number of repetitions; maximum number of motifs, 15; and, the optimum width of each motif, between six and 100 residues. The MAST program (http://meme-suite.org/tools/mast) was also used to search detected motifs in protein databases.

### Gene expression analysis

Global gene expression profiles over various tissues were investigated based on Illumina pair-ended RNA-seq samples, that is, expanding leaf (SAMEA1034168), developing male flower (SAMEA1034167), developing endosperm II/III (SAMEA1034165), developing endosperm V/VI (SAMEA1034166), developing seed (SAMN02688010), and germinating seed (SAMEA1034164) in castor; root (SAMN03160709), leafage (half expanded leaf, SAMN03160710), expanded leaf (SAMN03486846), flower bud (SAMN03733282), and seed (SAMN03160711) in physic nut. Expression profiles of JcDof genes during flower development were examined over five typical stages, that is, IND (undifferentiated inflorescence of 0.5 cm diameter, SAMN07525083), PID1 (carpel primordia beginning to differentiate, SAMN07525085), PID2 (three distinct carpels formed, SAMN07525084), STD1 (stamen primordia beginning to differentiate, SAMN07525082), and STD2 (10 complete stamens formed, SAMN07525086). JcDof genes involved in *Colletotrichum gloeosporioides* response were identified using SAMN02905749, SAMN02905750, SAMN02905751, and SAMN02905752. The filtered reads were mapped to the coding sequences (CDS) of Dof and other protein-coding genes using Bowtie 2 (Langmead & Salzberg, 2012), and the fragments per kilobase of exon per million fragments mapped (FPKM) method (Mortazavi et al., 2008) was used for the determination of transcript levels. To analyze differentially expressed genes (DEGs), the parameters “FDR< 0.001” and “log_2_Ratio ≥1” were used. Unless stated otherwise, the tools used in this study were performed with default parameters.

## Results

### Characterization of 25 Dof family genes in physic nut

**Figure 1:**
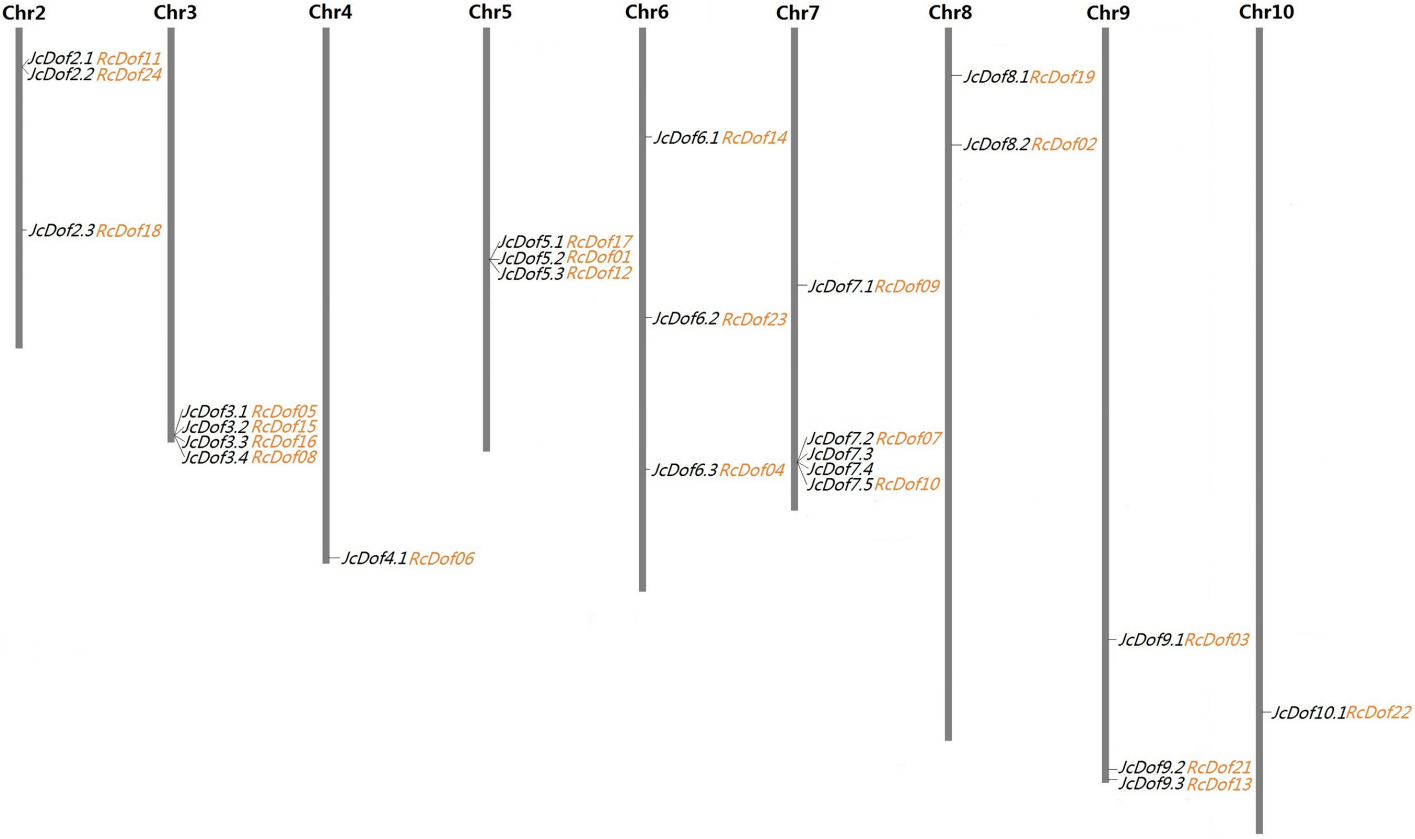
Chromosomal locations of 25 JcDof genes and their collinear genes in castor. Shown are nine chromosomes encoding Dof genes and the chromosome serial number is indicated at the top of each chromosome. Twenty-three RcDof genes shown just behind their collinear genes in physic nut were marked in orange.

As shown in Table 1, the search of the physic nut genome resulted in 25 Dof-coding loci from 16 out of the 6,023 scaffolds. Compared with the original genome annotation (Wu et al., 2015), one more locus was identified from scaffold241 (denoted *JcDof6.1*, see File S1). Interestingly, this gene was also identified by Wang et al. (2018), however, *JcDof7.4*, another member encoded by scaffold341, was not reported in their study. Although most scaffolds harbor a single Dof gene, four of them were found to contain more than one, that is, scaffold341 (4), scaffold684 (4), scaffold328 (3), and scaffold18 (2) (Table 1). Based on available genetic markers (Wu et al., 2015), these scaffolds can be further anchored to nine out of the 11 chromosomes (Chrs), only excluding Chromosomes 1 and 11. The gene distribution looks uneven: Chromosomes 4 and 10 contain a single Dof gene, whereas Chromosome 7 harbors the most of five (Fig. 1).

**Table 1:** Dof family genes identified in this study.

Gene name	Other name	Locus ID	Scaffold location	Chr	Nucleotide length (bp, from start to stop codons)		Intron no.	EST no.	AA	MW (kDa)	*p*I	GRAVY	Dof location	Comment
					CDS	Gene								
*JcDof2.1*	*JcDof-19*^a^	JCGZ_24801	scaffold84:2295234–2297144	Chr2	750	750	0	0	249	26.50	8.26	−0.872	18–76	
*JcDof2.2*	*JcDof-22*^a^	JCGZ_05689	scaffold18:3188247–3190056	Chr2	948	1,141	1	0	315	33.92	9.23	−0.650	71–129	
*JcDof2.3*	*JcDof-23*^a^	JCGZ_05589	scaffold18:2233935–2231886	Chr2	873	1,752	1	0	290	32.43	6.65	−0.881	36–94	
*JcDof3.1*	*JcDof-1*^a^	JCGZ_21565	scaffold684:2303230–2301983	Chr3	483	601	1	0	160	18.22	9.40	−0.946	40–98	
*JcDof3.2*	*JcDof-2*^a^	JCGZ_21459	scaffold684:1710168–1707923	Chr3	1,098	1,284	1	2	365	40.04	8.72	−0.698	67–125	
*JcDof3.3*	*JcDof-3*^a^	JCGZ_21458	scaffold684:1698897–1701026	Chr3	966	966	0	3	321	34.86	9.42	−0.926	39–97	
*JcDof3.4*	*JcDof-4*^a^	JCGZ_21405	scaffold684:1387777–1386435	Chr3	852	852	0	1	283	31.23	8.87	−0.692	48–106	
*JcDof4.1*	*JcDof-17*^a^	JCGZ_04337	scaffold159:487148–485559	Chr4	738	738	0	0	245	25.88	8.52	−0.600	21–79	
*JcDof5.1*	*JcDof-11*^a^	JCGZ_11472	scaffold328:1102402–1100148	Chr5	939	1,507	1	3	312	34.99	6.75	−0.791	52–110	
*JcDof5.2*	*JcDof-10*^a^	JCGZ_11638	scaffold328:2164470–2167731	Chr5	1,416	2,505	1	1	471	51.49	6.61	−0.889	121–179	
*JcDof5.3*	*JcDof-9*^a^	JCGZ_11766	scaffold328:3140516–3138621	Chr5	1,005	1,005	0	5	334	36.53	6.86	−0.891	31–89	
*JcDof6.1*	*JcDof-16*^a^	–	scaffold241:416184–417205	Chr6	849	849	0	0	282	30.89	5.14	−0.658	10–68	Not annotated
*JcDof6.2*	*JcDof-15*^a^	JCGZ_09056	scaffold250:1824207–1825861	Chr6	1,062	1,220	1	0	353	36.58	9.14	−0.486	75–133	Misannotated
*JcDof6.3*	*JcDof-18*^a^	JCGZ_02656	scaffold122:901889–898542	Chr6	1,494	2,560	1	2	497	53.63	7.78	−0.648	101–159	
*JcDof7.1*	*JcDof-8*^a^	JCGZ_18734	scaffold537:1159296–1157885	Chr7	1,035	1,035	0	3	344	36.72	8.97	−0.691	31–89	
*JcDof7.2*	*JcDof-14*^a^	JCGZ_12316	scaffold341:636392–637943	Chr7	741	741	0	0	246	25.33	8.39	−0.429	26–84	Misannotated
*JcDof7.3*	*JcDof-12*^a^	JCGZ_12318	scaffold341:649477–650499	Chr7	702	702	0	0	233	24.31	9.44	−0.295	26–84	Misannotated
*JcDof7.4*	*–*	JCGZ_12319	scaffold341:656395–657281	Chr7	741	741	0	0	246	25.23	8.61	−0.380	26–84	Misannotated
*JcDof7.5*	*JcDof-13*^a^	JCGZ_12335	scaffold341:727650–729095	Chr7	981	981	0	0	326	34.90	9.08	−0.547	63–121	
*JcDof8.1*	*JcDof-7*^a^	JCGZ_14231	scaffold392:2131890–2129929	Chr8	864	1375	1	0	287	31.28	8.87	−0.727	38–96	
*JcDof8.2*	*JcDof-20*^a^	JCGZ_15619	scaffold46:334880–337509	Chr8	1,398	2,134	1	0	465	51.09	6.80	−0.958	129–187	
*JcDof9.1*	*JcDof-6*^a^	JCGZ_19859	scaffold617:156090–159706	Chr9	1,557	2,966	1	1	518	55.72	5.88	−0.895	148–206	
*JcDof9.2*	*JcDof-5*^a^	JCGZ_20398	scaffold660:392825–390625	Chr9	909	1,369	1	3	302	32.79	8.69	−0.695	39–97	
*JcDof9.3*	*JcDof-21*^a^	JCGZ_25217	scaffold86:715786–716941	Chr9	864	864	0	1	287	32.76	4.65	−0.668	28–86	
*JcDof10.1*	*JcDof-24*^a^	JCGZ_01467	scaffold11:2547559–2545613	Chr10	1,017	1,237	1	1	338	35.69	9.19	−0.500	70–128	
*RcDof01*	*RcDof-17*^b^	30138.t000239	scaffold30138:1637304–1634642	–	1,467	2,663	1	5	488	53.04	6.15	−0.802	129–187	
*RcDof02*	*–*	29813.t000055	scaffold29813:341568–343736	–	1,473	2,269	1	0	490	53.61	6.61	−0.814	138–196	Misannotated
*RcDof03*	*RcDof-3*^b^	28838.t000008	scaffold28838:42548–39673	–	1,494	2,876	1	1	497	53.91	5.85	−0.894	136–194	
*RcDof04*	*RcDof-14*^b^	30072.t000004	scaffold30072:31747–29003	–	1,521	2,745	1	0	506	54.98	6.05	−0.732	110–168	
*RcDof05*	*RcDof-11*^b^	29900.t000086	scaffold29900:537300–536854	–	489	897	1	1	162	18.46	9.10	−0.909	36–94	Misannotated
*RcDof06*	*RcDof-19*^b^	30170.t000353	scaffold30170:1886017–1884911	–	744	744	0	0	247	26.47	6.41	−0.637	25–83	Misannotated
*RcDof07*	*RcDof-7*^b^	29676.t000058	scaffold29676:352074–351337	–	738	738	0	1	245	25.38	8.44	−0.381	28–86	
*RcDof08*	*RcDof-10*^b^	29851.t000017	scaffold29851:83027–82104	–	924	924	0	1	307	34.02	9.23	−0.727	45–103	
*RcDof09*	*RcDof-4*^b^	29235.t000001	scaffold29235:11382–12419	–	1,038	1,038	0	2	345	36.86	9.02	−0.655	34–92	
*RcDof10*	*RcDof-6*^b^	29676.t000039	scaffold29676:263441–262434	–	1,008	1,008	0	0	335	35.63	8.76	−0.461	66–124	
*RcDof11*	*RcDof-1*^b^	27395.t000003	scaffold27395:18038–17286	–	753	753	0	1	250	26.59	7.59	−0.939	18–76	
*RcDof12*	*RcDof-16*^b^	30138.t000092	scaffold30138:654539–655480	–	942	942	0	3	313	34.14	6.88	−0.727	32–90	
*RcDof13*	*RcDof-2*^b^	27985.t000022	scaffold27985:152767–153651	–	912	912	0	0	303	33.50	4.48	−0.522	29–87	Misannotated
*RcDof14*	*RcDof-5*^b^	29333.t000010	scaffold29333:68913–67987	–	927	927	0	0	308	33.79	6.13	−0.706	10–68	
*RcDof15*	*RcDof-20*^b^	30190.t000567	scaffold30190:261178–262436	–	1,074	1,259	1	1	357	39.30	8.77	−0.722	54–112	
*RcDof16*	*RcDof-21*^b^	30190.t000570	scaffold30190:276489–275545	–	945	945	0	5	314	33.56	9.51	−0.722	26–84	
*RcDof17*	*RcDof-18*^b^	30147.t000216	scaffold30147:3515510–3513099	–	1,005	1,656	1	0	334	37.33	6.71	−0.784	52–110	Misannotated
*RcDof18*	*RcDof-8*^b^	29688.t000002	scaffold29688:19134–18280	–	902	1,789	1	0	302	33.49	6.81	−0.803	36–79	Misannotated
*RcDof19*	*RcDof-9*^b^	29742.t000016	scaffold29742:85032–84102	–	924	1,576	1	0	307	33.20	8.44	−0.639	36–79	Misannotated
*RcDof20*	*RcDof-15*^b^	30098.t000004	scaffold30098:40138–41558	–	897	1,421	1	0	298	32.86	9.00	−0.715	40–98	
*RcDof21*	*RcDof-13*^b^	30068.t000036	scaffold30068:226227–227663	–	921	1,437	1	0	306	32.97	8.64	−0.660	44–102	
*RcDof22*	*RcDof-12*^b^	30003.t000001	scaffold30003:4571–5770	–	1,017	1,200	1	0	338	35.71	9.35	−0.612	77–135	
*RcDof23*	*–*	30025.t000009	scaffold30025:81058–82256	–	1,065	1,253	1	0	354	37.21	9.26	−0.531	84–142	Misannotated
*RcDof24*	*–*	28986.t000003	scaffold28986:16337–17513	–	933	1,152	1	0	310	33.42	9.48	−0.650	72–130	Misannotated

**Notes:**

a Gene names proposed by Wang et al. (2018).

b Gene names proposed by Jin, Chandrasekaran & Liu (2014).

Homology search showed that 12 JcDof genes have EST hits in GenBank, that is, *JcDof3.2*, *JcDof3.3*, *JcDof3.4*, *JcDof5.1*, *JcDof5.2*, *JcDof5.3*, *JcDof6.3*, *JcDof7.1*, *JcDof9.1*, *JcDof9.2*, *JcDof9.3*, and *JcDof10.1*, where *JcDof5.3* harbors maximum of five hits (Table 1). The full-length cDNA sequences of *JcDof3.2* (i.e., FJ605173), *JcDof5.1* (i.e., KF022019), *JcDof5.2* (i.e., GQ256647; *JcDof1*), *JcDof7.2* (i.e., KF022024; *JcDof2*), and *JcDof9.1* (i.e., GQ256648 and KF022017; *JcDof3*) have also been reported in GenBank. Moreover, the expression of other family members was supported by RNA-seq reads derived from various tissue transcriptomes, including callus, root, leaf, flower, inflorescence meristem, seed, and embryo. Based on read alignment, the transcription regions of all JcDof genes were extended and four predicted gene models were also optimized (Table 1; Files S2–S5). The CDS of three genes (i.e., *JcDof7.2*, *JcDof7.3*, and *JcDof7.4*), which are located on scaffold341, were shown to exhibit relatively high identity, that is, 87.7% between *JcDof7.2* and *JcDof7.3*, 87.4% between *JcDof7.3* and *JcDof7.4*, and 97.4% between *JcDof7.2* and *JcDof7.4*. They were defined as tandem duplications for their adjacent organization (Lee et al., 2013).

### Characterization of 24 Dof family genes in castor

A total of 24 Dof-coding loci were identified from 21 castor scaffolds (Table 1). As observed in physic nut, most of these castor scaffolds contain a single Dof gene, whereas scaffold30138, scaffold30190, and scaffold29676 harbor two instead. The expression of all these genes was supported by available ESTs and/or RNA-seq reads. Compared with the automatic genome annotation (Chan et al., 2010), nine predicted gene models were manually optimized based on read alignment (see Files S6–S14). Conserved microsynteny was observed between castor and physic nut Dof-coding scaffolds. Although the current castor genome is fragmented in 25,763 scaffolds, 23 out of the 24 RcDof genes could be anchored to nine physic nut chromosomes based on synteny analysis (Fig. 1).

### Phylogenetic analysis and definition of orthologous groups

**Figure 2:**
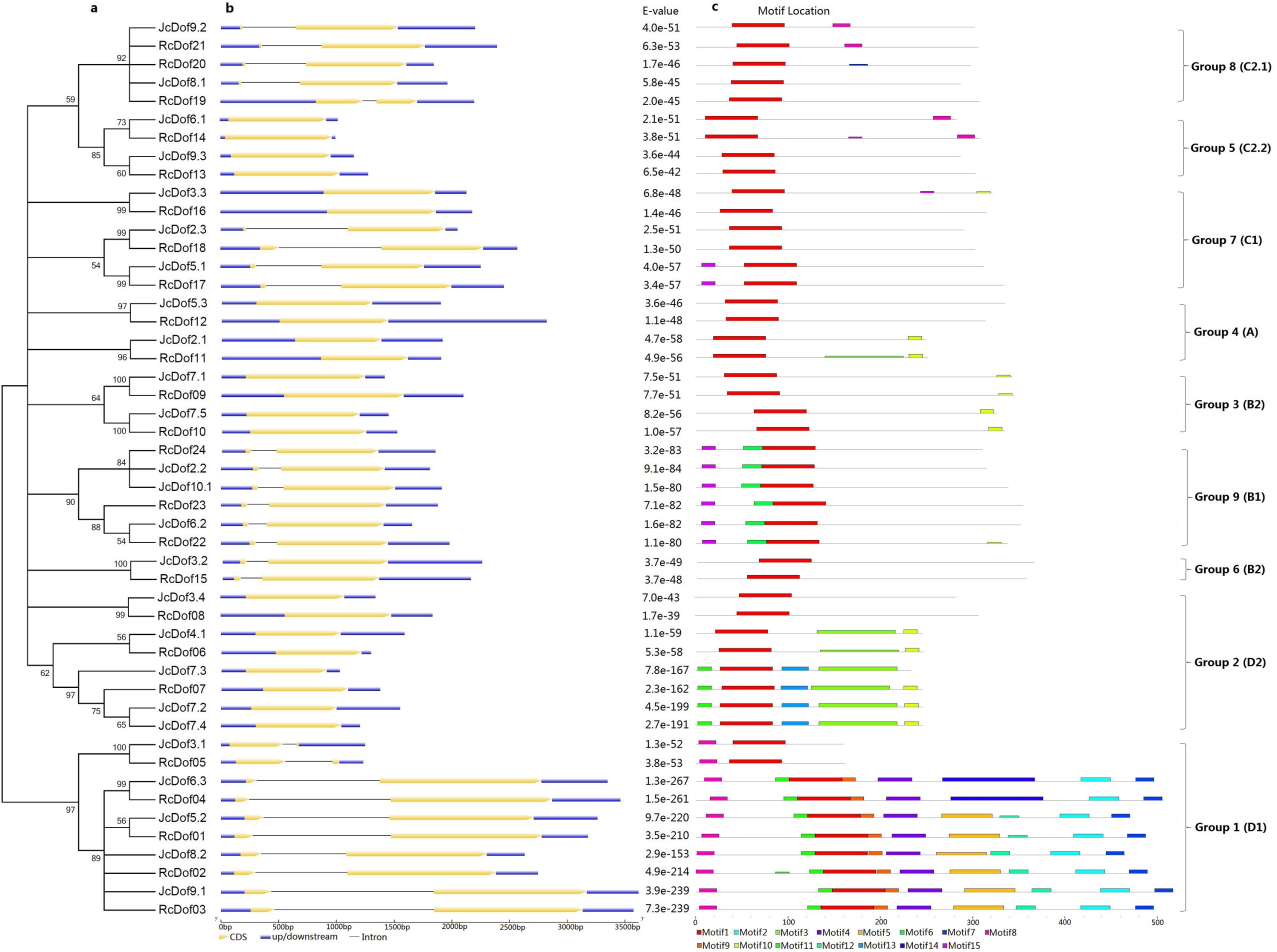
Structural and phylogenetic analysis of physic nut and castor Dof genes. (A) The unrooted phylogenetic tree resulting from full-length Dof proteins with MEGA6, where the distance scale denotes the number of amino acid substitutions per site. (B) The graphic representation of exon-intron structures displayed using GSDS. (C) The distribution of conserved motifs among Dof proteins, where different motifs are represented by different color blocks as indicated at the bottom of the figure and the same color block in different proteins indicates a certain motif.

To reveal the evolutionary relationships of physic nut and castor Dof genes, an unrooted phylogenetic tree was constructed using full-length Dof proteins. As shown in Fig. 2, 49 Dofs are split into nine groups representing four previously defined subfamilies (i.e., A, B, C, and D): Groups 1, 2, 4, 5, 7, 8, and 9 correspond to D1, D2, A, C2.2, C1, C2.1, or B1, whereas Groups 3 and 6 correspond to B2 as described in *Arabidopsis* (Lijavetzky, Carbonero & Vicente-Carbajosa, 2003). The classification is supported by analysis of exon-intron structures and conserved motifs as shown below.

To facilitate the transfer of functional information obtained in model plants, orthologs of Jc/RcDofs were further identified in *Arabidopsis* and rice, two well-studied species of dicotyledons or monocotyledons respectively. The detailed orthologous relationships are shown in Table 2, whereas 24 orthologous groups (OGs) were also proposed based on the definition of a certain OG with at least one member found in at least two out of four examined species. Genes whose functions have been characterized were also summarized in each OG that they belong. Nine phylogenetic groups identified above were shown to contain one to five OGs. Consistent with the phylogenetic analysis, species-specific gene loss was observed and several OGs were only found in physic nut and castor (i.e., OG-1b and OG-2c) (Table 2).

**Table 2:** 24 Dof OGs based on comparison of four representative species.

OG	Castor	Physic nut	Arabidopsis	Rice	Function
1a	*RcDof01*	*JcDof5.2*	*AtDof3.3*	*OsDof3.1*	AtDof3.3 (CDF3), AtDof5.5 (CDF1), photoperiodic flowering (Fornara et al., 2009); OsDof3.1 (OsDof12), photoperiodic flowering (Li et al., 2009), and plant architecture formation (Wu et al., 2015)
			*AtDof5.5*	*OsDof10.1*	
1b	*RcDof02*	*JcDof8.2*	–	–	
1c	*RcDof03*	*JcDof9.1*	*AtDof5.2*	–	AtDof5.2 (CDF2), photoperiodic flowering (Fornara et al., 2009)
1d	*RcDof04*	*JcDof6.3*	–	*OsDof1.2*	
				*OsDof7.3*	
1e	*RcDof05*	*JcDof3.1*	*AtDof1.5*	*OsDof1.4*	AtDof1.5 (COG1), phytochrome signal (Park et al., 2003)
			*AtDof2.3*	*OsDof1.5*	
2a	*RcDof06*	*JcDof4.1*	*AtDof1.6*	–	
2b	*RcDof07*	*JcDof7.2*	*AtDof3.4*	–	AtDof3.4 (OBP1), defence response (Zhang et al., 1995), and cell cycle regulation (Skirycz et al., 2008); AtDof5.8 (SCAP1), vascular development (Konishi & Yanagisawa, 2007), stomatal functioning and morphogenesis (Negi et al., 2013)
		*JcDof7.3*	*AtDof5.8*		
		*JcDof7.4*			
2c	*RcDof08*	*JcDof3.4*	–	–	
3a	*RcDof09*	*JcDof7.1*	*AtDof5.7*	–	AtDof4.7, floral organ abscission deficiency (Wei et al., 2010)
			*AtDof4.7*		
3b	*RcDof10*	*JcDof7.5*	–	–	
4a	*RcDof11*	*JcDof2.1*	*AtDof1.7*	–	AtDof1.7 (DOF1), regulation of carbon skeleton production, nitrogen assimilation and reduction of glucose level (Yanagisawa et al., 2004)
			*AtDof3.1*		
4b	*RcDof12*	*JcDof5.3*	*AtDof5.4*	*OsDof2.4*	AtDof5.4 (OBP4), negative regulating cell cycle progression and cell growth (Xu et al., 2016), controling root growth and callus formation (Ramirez-Parra et al., 2017; Rymen et al., 2017)
				*OsDof6.1*	
5a	*RcDof13*	*JcDof9.3*	*AtDof1.2*	*OsDof5.2*	
5b	*RcDof14*	*JcDof6.1*	*AtDof3.5*	*OsDof1.6*	
6	*RcDof15*	*JcDof3.2*	*AtDof1.4*	*OsDof7.2*	
7c	*RcDof16*	*JcDof3.3*	–	–	
7a	*RcDof17*	*JcDof5.1*	*AtDof5.6*	*OsDof2.3*	AtDof5.6 (HCA2), interfascicular cambium formation and vascular tissue development (Guo et al., 2009)
7b	*RcDof18*	*JcDof2.3*	*AtDof2.1*	–	AtDof3.2 (DOF6), negatively affecting germination in non-after-ripened seeds (Rueda-Romero et al., 2012)
			*AtDof3.2*		
			*AtDof5.3*		
8a	*RcDof19*	*JcDof8.1*	*AtDof2.5*	–	AtDof2.5 (DAG2), seed germination (Gabriele et al., 2010); AtDof3.7 (DAG1, BBFa), seed germination (Papi et al., 2000, 2002; Gabriele et al., 2010)
			*AtDof3.7*		
8b	*RcDof20*	–	*AtDof4.1*	–	AtDof4.1 (ITD1), selective intercellular trafficking (Chen et al., 2013)
8c	*RcDof21*	*JcDof9.2*	*AtDof4.6*	*OsDof2.2*	OsDof4.1 (OsDOF18), controlling ammonium uptake (Wu et al., 2017)
				*OsDof4.1*	
9a	*RcDof22*	*JcDof10.1*	*AtDof5.1*	*OsDof3.6*	AtDof5.1, leaf axial patterning (Kim et al., 2010)
				*OsDof7.1*	
9b	*RcDof23*	*JcDof6.2*	*AtDof2.4*	–	AtDof2.4, vascular development (Konishi & Yanagisawa, 2007); AtDof3.6 (OBP3), phytochrome and cytochrome signaling (Kang et al., 2003; Ward et al., 2005)
			*AtDof3.6*		
9c	*RcDof24*	*JcDof2.2*	*AtDof1.1*	–	AtDof1.1 (OBP2), cell cycle regulation and glucosinolate biosynthesis (Skirycz et al., 2006, 2008)
			*AtDof2.2*		

### Exon-intron structures, sequence features, and conserved motifs

The exon-intron structure was analyzed based on optimized gene models and results showed that these genes contain no or a single intron (Table 1). Approximately 48.00% of JcDof genes and 43.48% of RcDof genes are intronless. Similar exon-intron structure was also observed in rice and *Arabidopsis*, though two-intron-containing genes (i.e., *AtDof3.6* and *AtDof3.7*, accounting for 5.6%) were also found in *Arabidopsis* (Table S1). Generally, the exon-intron structure is highly conserved within a certain phylogenetic group especially within a certain OG: Groups 2–5 usually contain no intron, whereas Groups 1 and 6–9 feature one intron with the exception of OG-7c.

**Figure 3:**
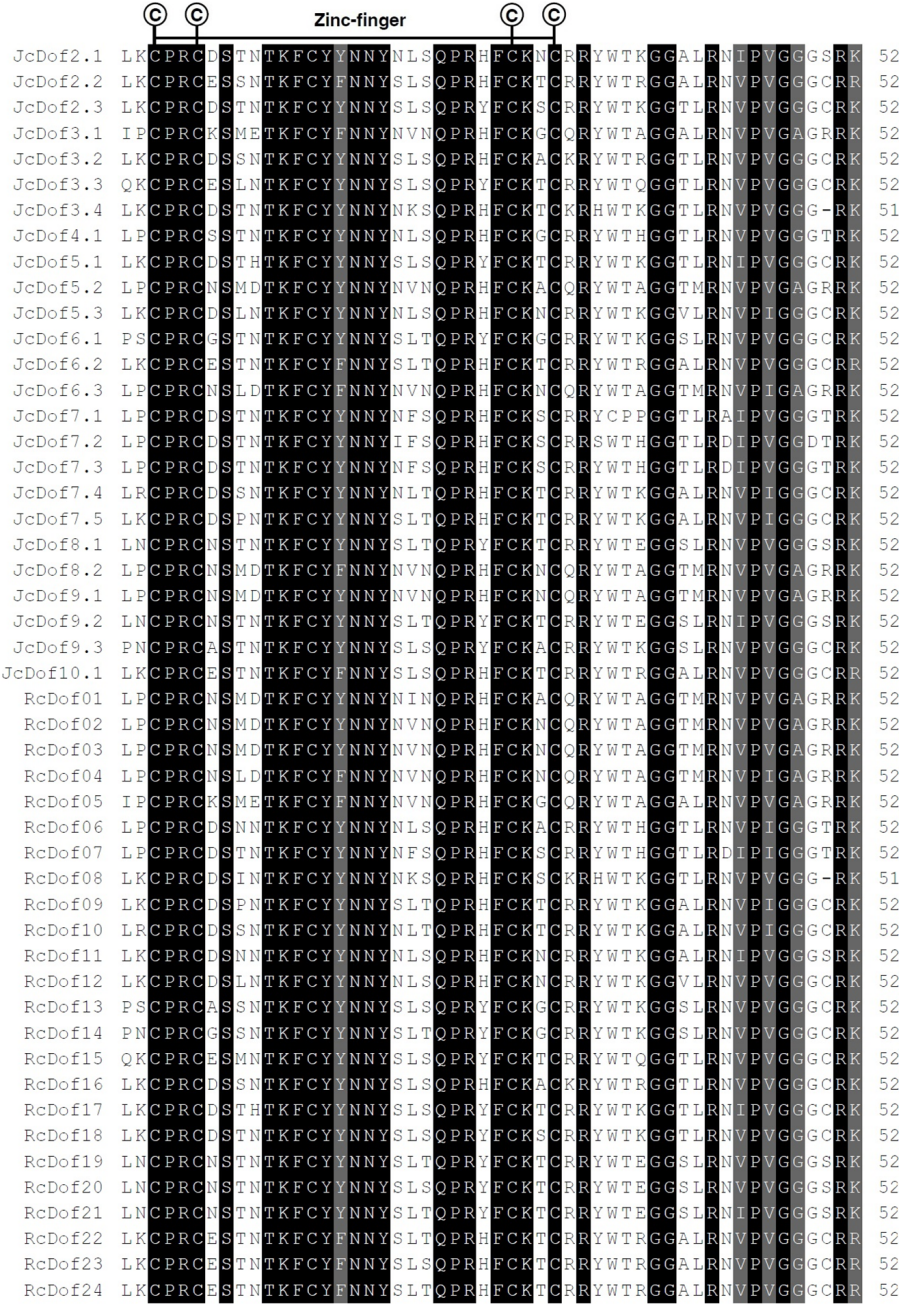
Dof domain sequence alignment of physic nut and castor Dof proteins. The four cysteine residues putatively responsible of the zinc-finger structure are indicated. Identical and similar amino acids are highlighted in black or dark gray, respectively.

Deduced JcDof and RcDof proteins consist of 160–518 or 162–506 amino acid residues, respectively. The MW ranges from 18.22 to 55.72 kDa for JcDofs, or from 18.46 to 54.98 kDa for RcDofs. The predicted *p*I value ranges from 4.65 to 9.44 for JcDofs, or from 4.48 to 9.51 for RcDofs. Nevertheless, the average protein length, MW and *p*I value are similar between two species, that is, 323 residues, 34.92 kDa or 8.00 in physic nut; 334 residues, 36.23 kDa or 7.78 in castor. The GRAVY value of all Dof proteins is less than 0, indicating their hydrophilic feature. All these proteins were shown to harbor a single Dof domain with the Cx_2_Cx_21_Cx_2_C zinc finger pattern, and were predicted to localize to the nucleus (Fig. 3).

Conserved motifs were also analyzed using MEME and results were shown in Fig. 2 and Fig. S1. Among 15 motifs identified, the broadly distributed Motif 1 is characterized as the Dof domain, in contrast, little is known about other motifs: Motif 8 is present in all members of Group 1, and several members of Groups 5 and 8; Motif 10 is widely present in Group 2, and several members of Groups 3 and 4; Motif 11 is present in all members of Group 1, and several members of Group 2; Motif 15 is present in all members of Group 9, and several members of Group 7; Motifs 2, 4, 5, 7, 9, 12 is limited to Group 1; Motifs 3 and 13 are limited to Group 2; Motif 6 is limited to Group 9; and, Motif 14 is limited to OG-1d of Group 1 (Fig. 2).

Gain or loss of certain motifs was observed within a certain group even between different OGs in the same group. Group 1 usually contains Motifs 8, 11, 1, 9, 4, 5, 12, 2, and 7 in orders; however, OG-1a only harbors Motifs 8 and 1, suggesting their early divergence; moreover, OG-1d harbors Motif 14 instead of Motifs 5 and 12 found in other OGs. OG-2b of Group 2 usually contains Motifs 11, 1, 13, 3, 10 in orders, in contrast, OG-2c only harbors Motif 1 and OG-2a has Motifs 1, 3, and 10, supporting this group can be further divided into three subgroups. Interestingly, JcDof7.3 has also lost Motif 10. OG-4b of Group 4 contains Motifs 1 and 10, whereas OG-4a only harbors Motif 1, supporting this group can be subdivided into two subgroups. OG-5b of Group 5 contains Motifs 1 and 8, whereas OG-5a only harbors Motif 1, supporting this group can also be subdivided into two subgroups. Group 8 usually features a single motif; however, OG-8c gained Motif 8 (Fig. 2).

### Expression profiles of JcDof genes

**Figure 4:**
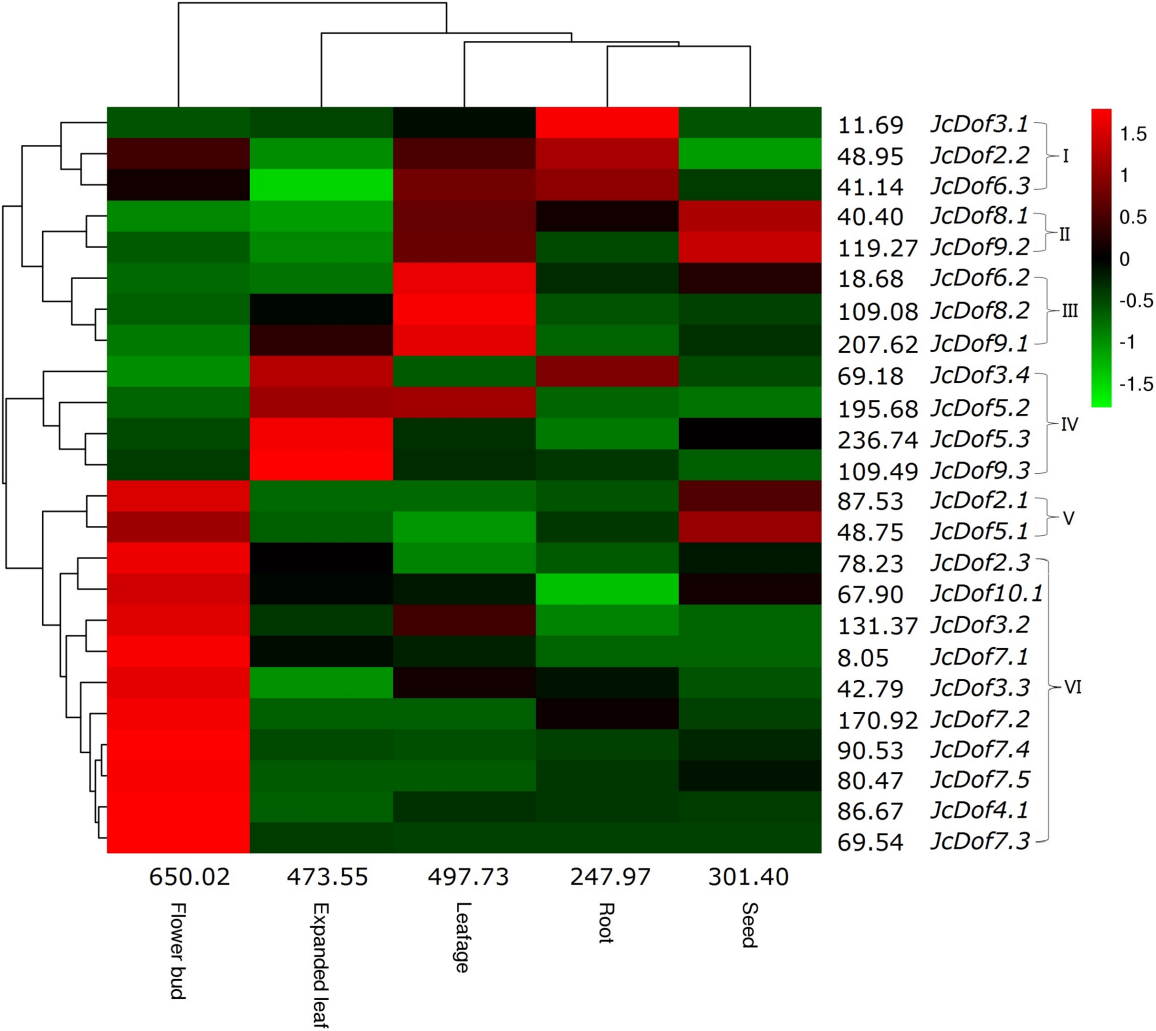
Tissue-specific expression profiles of JcDof genes. Color scale represents FPKM normalized log_10_ transformed counts where green indicates low expression and red indicates high expression.

Despite the expression of all JcDof genes, transcriptional profiling showed that *JcDof6.1* was lowly expressed in all tissues examined. Based on the FPKM value, the total transcripts of JcDof genes were shown to be most abundant in flower bud, moderate in expanded leaf and leafage, and relatively low in seed and root. Most genes were expressed in all five examined tissues, though their transcript levels are variable. According to the expression pattern, these genes can be divided into five main clusters: Cluster I is preferentially expressed in root, where *JcDof2.2* and *JcDof6.3* were also abundant in leafage and flower bud; Cluster II is predominantly expressed in seed as well as high abundance in leafage; Cluster III is mostly expressed in leafage, where *JcDof8.2* and *JcDof9.1* were also abundant in expanded leaf; Cluster IV is typically expressed in expanded leaf, where *JcDof3.4* and *JcDof5.2* were also abundant in root or leafage respectively; Clusters V and VI are mostly expressed in flower bud, where Cluster V members were also highly expressed in seed (Fig. 4). Several key family members were also identified in a certain tissue: *JcDof7.2* represents the most expressed gene in both flower bud and root; *JcDof5.3* represents the most expressed gene in both expanded leaf and seed; and, *JcDof9.1* represents the most expressed gene in leafage.

**Figure 5:**
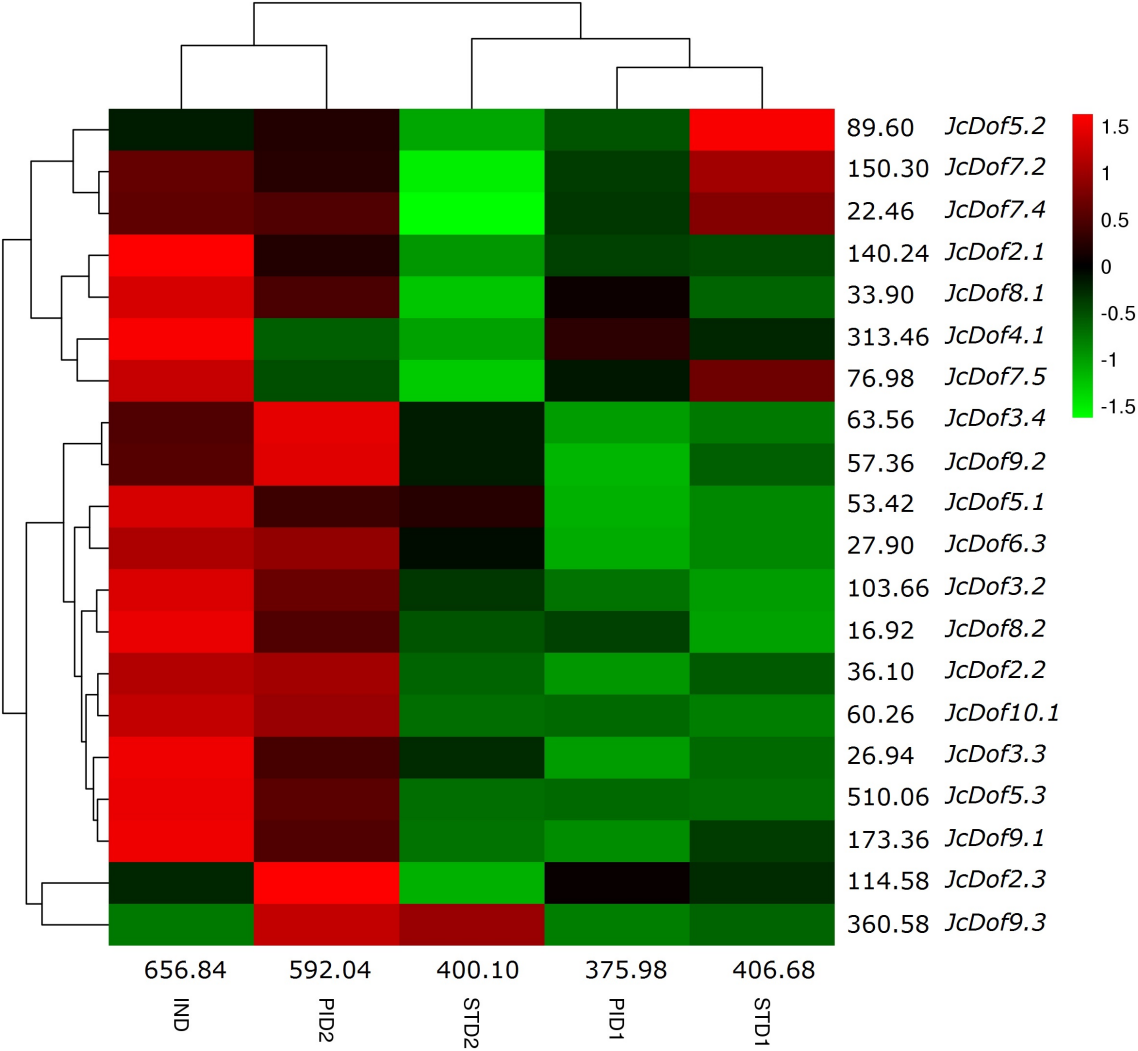
Expression profiles of JcDof genes over five typical flower stages. Color scale represents FPKM normalized log_10_ transformed counts where green indicates low expression and red indicates high expression.

Low seed yield, which was caused by the lower number of female flowers, is a bottleneck for commercial-scale exploration of physic nut. Transcriptome profile analysis of five typical stages during floral sex differentiation revealed thousands of DEGs involved in ovule and stamen development (Hui et al., 2017). With a focus on Dof family genes, we found that the total transcripts were most abundant in IND, followed by PID2, STD1, STD2, and PID1 (Fig. 5). Compared with IND, eight genes were significantly downregulated in PID1; three genes were significantly regulated in PID2, including one upregulated and two downregulated; 10 genes were significantly downregulated in STD1; 11 genes were significantly regulated in STD2, including one upregulated and 10 downregulated. Compared with PID1, nine genes were significantly regulated in PID2, including one downregulated and eight upregulated. Compared with STD1, six genes were significantly regulated in STD2, including two upregulated and four downregulated (Fig. 5; Table S2). More remarkable, the expression pattern of *JcDof9.3* suggests its key role in the later stage of both ovule and stamen development.

**Figure 6:**
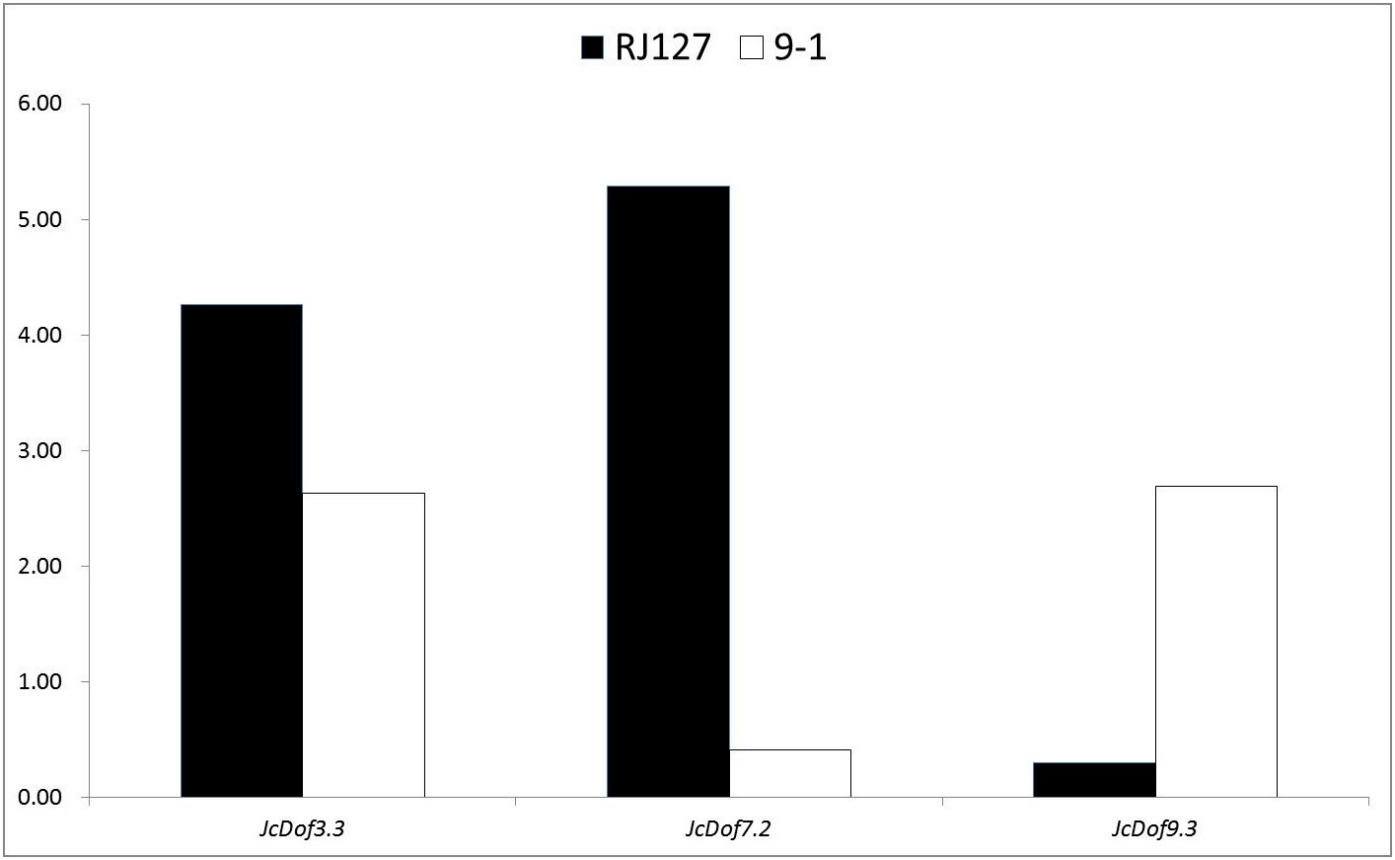
Fold change of JcDof genes involved in *C. gloeosporioides* response.

Anthracnose, caused by *C. gloeosporioides*, is an important disease causing damages on physic nut leaves, stems, and fruits (Ferreira-Pinto, Silva & Santos, 2011). To analyze the response of JcDof genes to this pathogen, two different genotypes (i.e., the susceptible RJ127 and the resistant 9-1) were artificially infected with pure isolate of *C. gloeosporioides*, and the induced leaves were collected at 2, 24, 96, and 144 h after infection and pooled for Illumina RNA-seq. Differential expression analysis showed that three JcDof genes were significantly regulated, that is, *JcDof3.3*, *JcDof7.2*, and *JcDof9.3*: *JcDof3.3* was upregulated in both cultivars, whereas *JcDof7.2* and *JcDof9.3* exhibit an opposite trend (Fig. 6), implying something different regulation mechanism of these two cultivars.

### Tissue-specific expression profiles of *RcDof* genes

**Figure 7:**
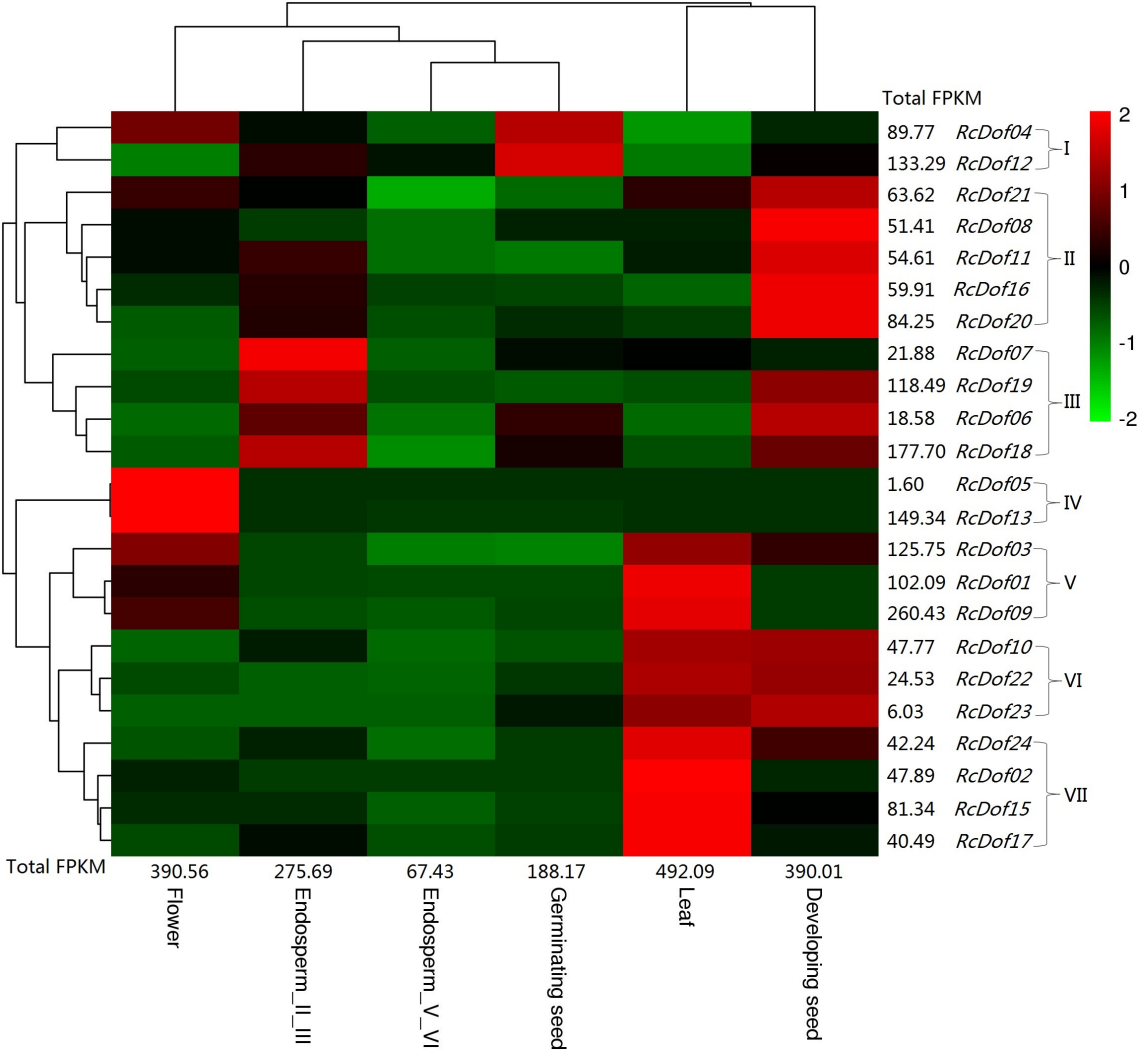
Tissue-specific expression profiles of RcDof genes. Color scale represents FPKM normalized log_10_ transformed counts where green indicates low expression and red indicates high expression.

Similar to its ortholog in physic nut (i.e., *JcDof6.1*), the transcript level of *RcDof14* was extremely low in all examined tissues, that is, leaf, male flower, endosperm I/III, endosperm V/VI, developing seed, and germinating seed. The total transcripts were most abundant in leaf (Class I); followed by male flower and developing seed (Class II, accounting for 79.3–79.4% of Class I); moderate in endosperm I/III and germinating seed (Class III, accounting for 38.2–56.0% of Class I); and, relatively low in endosperm V/VI (Class IV, accounting for 13.7% of Class I). Based on their expression patterns, these genes were grouped into seven main clusters: Cluster I is mostly expressed in germinating seed, including *RcDof04* and *RcDof12*, which were also abundant in male flower or endosperm I/III respectively; Cluster II is preferentially expressed in developing seed; Cluster III is predominantly expressed in endosperm I/III, where *RcDof19*, *RcDof06*, and *RcDof18* were also abundant in developing seed; Cluster IV is typically expressed in male flower; Cluster V is preferentially expressed in leaf and male flower; Cluster VI is preferentially expressed in leaf and developing seed; and, Cluster VII is preferentially expressed in leaf. As for a specific tissue, several key isoforms were also identified: *RcDof09* represents the most and second most expressed gene in leaf or male flower, respectively; *RcDof13* represents the most expressed gene in male flower; *RcDof19* represents the most expressed gene in both developing seed and endosperm I/III; *RcDof12* represents the most expressed gene in both germinating seed and endosperm V/VI; *RcDof18* represents the second most expressed genes in germinating seed, endosperm I/III and endosperm V/VI; *RcDof18* represents the second most expressed genes in developing seed (Fig. 7).

## Discussion

The importance of Dof transcription factors in plant-specific processes has prompted active research in model plants such as *Arabidopsis* and rice (Noguero et al., 2013; Gupta et al., 2015). By contrast, research in non-model plants is still limited. In physic nut and castor, two groups have attempted to identify Dof genes from the genomic level, which resulted in 24 or 21 family members, respectively (Jin, Chandrasekaran & Liu, 2014; Wang et al., 2018). However, due to the proteomes used in their studies are not well annotated, identification of the gene family seems to be far from complete. In the present study, the original genome sequences and various transcriptome data were also added in homology search. As expected, relatively more Dof genes, that is, 25 or 24, were identified from physic nut and castor genomes, respectively (Table 1). It is worth noting that, besides one gene (*JcDof6.1*) escaping from the original genome annotation, the gene models of 13 other genes were manually optimized based on available mRNA, that is, four in physic nut and nine in castor (Files S2–S14). Similar results were also observed when mining other gene families, for example, aquaporin, WRKY, papain-like cysteine protease, and respiratory burst oxidase homolog (Zou et al., 2015b, 2016a, 2016b, 2018; Zou, Yang & Zhang, 2019a), suggesting a manual curation of computationally predicted gene structures is highly needed in these two species. As for other non-model plants whose genomes were recently released, a similar expert revision is highly recommended.

The number of Dof family members in physic nut or castor is relatively smaller than most angiosperm plants reported thus far (Yanagisawa, 2002; Lijavetzky, Carbonero & Vicente-Carbajosa, 2003; Yang, Tuskan & Cheng, 2006; Ma et al., 2015; da Silva et al., 2016; Wu et al., 2016; Wen et al., 2016; Zou, Zhu & Zhang, 2019b). In fact, Dof proteins were characterized as a plant-specific transcription factor family that is present as a single member in unicellular algae (Moreno-Risueno et al., 2007), and the family members have highly expanded along with several WGD events, for example, the γ event for core eudicots and the τ event for monocots (Jiao et al., 2012, 2014). Moreover, *Arabidopsis* and rice experienced two more recent doubling events, named β and α, or σ and ρ, respectively (Bowers et al., 2003; Jiao et al., 2014). Compared with ancient WGDs, recent WGDs play a more important role in gene expansion due to limited time for gene elimination (Panchy, Lehti-Shiu & Shiu, 2016; Zou et al., 2016a, 2017a; Zou, Xie & Yang, 2017b; Zou, Zhu & Zhang, 2019b; Zou, Yang & Zhang, 2019a). Taking *Arabidopsis* for an example, a high number of AtDof genes were shown to result from recent gene duplication, that is, β WGD (8), α WGD (6), tandem duplication (2), and proximal duplication (1) (Wang, Tan & Paterson, 2013; Ma et al., 2015). By contrast, none or only two recent duplicates were found in castor and physic nut, respectively, which is consistent with no recent WGD occurred in these two species (Chan et al., 2010; Wu et al., 2015). Two duplicates identified in physic nut were both derived from tandem duplication, which is proven to play key roles in adaptation and generation of species-specific traits (Chan et al., 2010; Qian & Zhang, 2014; Tang et al., 2016).

Based on phylogenetic analysis, Dof genes in *Arabidopsis* and rice were previously divided into four subfamilies and 10 groups named A, B1, B2, C1, C2.1, C2.2, C3, D1, D2, and D3 (Lijavetzky, Carbonero & Vicente-Carbajosa, 2003). In that study, sequences of the conserved Dof domain were used, however, this strategy usually cannot resolve the evolutionary relationships of ancient and recent duplicates. Thereby, the full-length proteins were adopted in our study. Moreover, orthologs across different species including model plants *Arabidopsis* and rice were identified using BRH-based BLAST analysis (as discussed below), where the orthologous relationships between physic nut and castor were also confirmed by synteny analysis. Our results showed that, except for C3 and D3 that is specific to *Arabidopsis* or rice, respectively, most groups can be found in both physic nut and castor (Fig. S2). Moreover, B2 is obviously split into two clusters, which is also supported by exon-intron structures and conserved motifs (Fig. 2). Thereby, we updated the subclassification as Group 1 (D1), 2 (D2), 3 (B2), 4 (A), 5 (C2.2), 6 (B2), 7 (C1), 8 (C2.1), and 9 (B1). Among them, Group 1 represents the largest group not only in physic nut and castor but also in *Arabidopsis* and rice (Fig. S2).

Species-specific evolution of the Dof gene family in physic nut and castor was further investigated. Consistent with their taxonomic classification standing within the same plant family, a significant level of syntenic relation and nearly one-to-one orthologous relationship were observed between physic nut and castor Dof genes. The 25 JcDof genes are distributed across nine physic nut chromosomes, and 23 out of 24 RcDof genes could be anchored to these chromosomes based on synteny analysis. To infer putative functions of Jc/RcDof genes, 24 OGs were also proposed based on comparison of physic nut, castor, *Arabidopsis* and rice Dofs. Compared with castor, two tandem duplications (*JcDof7.2*/*JcDof7.3*/*JcDof7.4*) and one gene loss (the ortholog of *RcDof20*) were found in physic nut (Table 2). The evolution pattern is something different from the WRKY transcription factor gene family, where one gene loss, one intron loss and one proximal duplication were found in castor in comparison to physic nut (Zou et al., 2016b). Interestingly, several OGs have no members in *Arabidopsis* and/or rice, suggesting the occurrence of species-specific gene loss (Table 2).

Putative roles of Jc/RcDof genes can be inferred from their expression profiles and their orthologs in *Arabidopsis* and/or rice, whose functions have been characterized (Figs. 4–7; Table 2). Among nine phylogenetic groups identified, Group 1 includes the most of five OGs, where OG-1a and OG-1e are widely distributed. OG-1d, which is absent from *Arabidopsis*, is widely present in flowering plants including papaya (*Carica papaya*, i.e., evm.TU.supercontig_161.44), a species standing very close to *Arabidopsis*, supporting species-specific gene loss. OG-1c, which is absent from rice, may be dicot-specific, since it can be traced back to *Aquilegia coerulea*, a member of the basal-most eudicot clade (Sharma et al., 2014; Zou et al., 2018). OG-1b, which is absent from both *Arabidopsis* and rice, may appear in the last common ancestor of core eudicots along with the γ event, since it is present in eudicots but not *A. coerulea*. Thus far, four members of OG-1a (i.e., *AtDof5.5*, *AtDof3.3*, *OsDof3.1*, and *JcDof5.2*) and two members of OG-1c (i.e., *AtDof5.2* and *JcDof9.1*) were shown to be involved in photoperiodic control of flowering (Fornara et al., 2009; Li et al., 2009; Yang et al., 2011), where *AtDof3.3* is also associated with the tolerance to drought, cold and osmotic stresses (Corrales et al., 2017). *AtDof1.5*, a member of OG-1e, is involved in phytochrome signaling (Park et al., 2003), and *RcDof05* was shown to be upregulated by ABA in leaf (Jin, Chandrasekaran & Liu, 2014), implying their similar functions.

Group 2, which includes three OGs, may be dicot-specific, since they are widely present in eudicots but not monocots. OG-2b and OG-2c can also been found in *A. coerulea*, that is, Aqcoe5G171900 or Aqcoe4G144000, respectively, suggesting their early diversification. The absence of OG-2c from *Arabidopsis* may be due to species-specific gene loss, since it is also present in papaya (i.e., evm.TU.supercontig_1117.2). By contrast, OG-2a may be generated in core eudicots along with the γ event. Compared with castor, OG-2b has been highly expanded via tandem duplication in physic nut (Table 2). Thus far, two members of OG-2b, that is, *AtDof3.4* and *AtDof5.8*, have been characterized, which were shown to participate in regulation of cell size and number, vascular development, stomatal functioning and morphogenesis (Konishi & Yanagisawa, 2007; Skirycz et al., 2008; Negi et al., 2013).

Group 3 contains two OGs that can be traced back to *Amborella trichopoda*, the sister lineage to all other flowering plants (Amborella Genome Project, 2013), though species-specific loss of OG-3b was observed in *Arabidopsis* (Table 2). The sole gene characterized in this group, that is, *AtDof4.7* (a member of OG-3a), is associated with floral organ abscission deficiency (Wei et al., 2010).

Group 4 contains two OGs that are widely present in flowering plants including *A. trichopoda*. However, species-specific loss of OG-4a was observed in rice as well as a high number of other monocots. For two genes characterized in this group, *AtDof1.7* (a member of OG-4a) is associated with carbon skeleton production, nitrogen assimilation and reduction of glucose level (Yanagisawa et al., 2004), whereas *AtDof5.4* (a member of OG-4b) is involved in regulation of cell cycle progression, cell growth, root growth and callus formation (Xu et al., 2016; Ramirez-Parra et al., 2017; Rymen et al., 2017).

Group 7 contains three OGs that can be traced back to *A. trichopoda*. OG-7b, which is absent from rice, is widely present in monocots as well as *A. trichopoda* (i.e., evm_27.TU.AmTr_v1.0_scaffold00080.30). OG-7c, which is absent from *Arabidopsis*, is widely present in dicots as well as papaya (i.e., evm.TU.supercontig_464.1), suggesting species-specific gene loss. Among two genes characterized in this group, *AtDof5.6* (a member of OG-7a) is associated with interfascicular cambium formation and vascular tissue development (Guo et al., 2009), whereas *AtDof3.2* (a member of OG-7b) is involved in regulation of germination in non-after-ripened seeds (Rueda-Romero et al., 2012).

Group 8 includes three OGs: OG-8c is widely present in flowering plants including *A. trichopoda*, whereas OG-8a and OG-8b may be dicot-specific that appeared in core eudicots along with the γ event. *AtDof3.7* and *AtDof2.5*, two members of OG-8a, were shown to control seed germination via a maternal switch (Papi et al., 2000, 2002; Gabriele et al., 2010). *AtDof4.1*, a member of OG-8b, is associated with selective intercellular trafficking (Chen et al., 2013). *OsDof4.1*, a member of OG-8c, is involved in ammonium transport and nitrogen distribution (Wu et al., 2017).

Similar to Group 8, Group 9 can also be traced back to *A. trichopoda* and has expanded in core eudicots along with the γ event. Among three OGs identified, OG-9a is closest to monocot homologs. *AtDof5.1*, a member of OG-9a, is associated with leaf axial patterning (Kim et al., 2010). *AtDof2.4* and *AtDof3.6*, two members of OG-9b, are involved in vascular development, phytochrome and cytochrome signaling (Kang et al., 2003; Ward et al., 2005; Konishi & Yanagisawa, 2007). *AtDof1.1*, a member of OG-9c, is involved in cell cycle regulation and glucosinolate biosynthesis (Skirycz et al., 2006, 2008).

By contrast, little information is available for another two groups, that is, Group 5 (two OGs) and Group 6 (a single OG), though they are widely distributed (Table 2).

## Conclusion

Our paper presents the genome-wide comparative evolutionary analysis of Dof family genes in physic nut and castor, two economically important Euphorbiaceae plants that have not experienced any recent WGD. The family number of 25 JcDof or 24 RcDof genes is relatively smaller than that in other terrestrial plants reported thus far. Nevertheless, the family is highly diverse and nine groups representing previously defined four subfamilies were found. Moreover, among 24 OGs identified, several OGs were absent from *Arabidopsis* and/or rice, suggesting species-specific evolution and their specific functions in these two and/or other species. Conserved microsynteny and nearly one-to-one orthologous relationships were observed between JcDof and RcDof genes, though two tandem duplications and one gene loss were found in the physic nut Dof gene family. Furthermore, their expression profiles and putative roles were also investigated and discussed with functional information available in *Arabidopsis* and/or rice. These findings will not only facilitate further studies of Dof genes in physic nut and castor, but also provide a useful reference to analyze the Dof gene family in other species.

## Supplemental Information

10.7717/peerj.6354/supp-1 Supplemental Information 1 File S1. The gene model for JcDof6.1.

10.7717/peerj.6354/supp-2 Supplemental Information 2 File S2. The gene model for JcDof6.2.

10.7717/peerj.6354/supp-3 Supplemental Information 3 File S3. The gene model for JcDof7.2.

10.7717/peerj.6354/supp-4 Supplemental Information 4 File S4. The gene model for JcDof7.3.

10.7717/peerj.6354/supp-5 Supplemental Information 5 File S5. The gene model for JcDof7.4.

10.7717/peerj.6354/supp-6 Supplemental Information 6 File S6. The gene model for RcDof02.

10.7717/peerj.6354/supp-7 Supplemental Information 7 File S7. The gene model for RcDof05.

10.7717/peerj.6354/supp-8 Supplemental Information 8 File S8. The gene model for RcDof06.

10.7717/peerj.6354/supp-9 Supplemental Information 9 File S9. The gene model for RcDof13.

10.7717/peerj.6354/supp-10 Supplemental Information 10 File S10. The gene model for RcDof17.

10.7717/peerj.6354/supp-11 Supplemental Information 11 File S11. The gene model for RcDof18.

10.7717/peerj.6354/supp-12 Supplemental Information 12 File S12. The gene model for RcDof19.

10.7717/peerj.6354/supp-13 Supplemental Information 13 File S13. The gene model for RcDof23.

10.7717/peerj.6354/supp-14 Supplemental Information 14 File S14. The gene model for RcDof24.

10.7717/peerj.6354/supp-15 Supplemental Information 15 Table S1. Detailed information of *Dof* family genes in *Arabidopsis* and rice.

10.7717/peerj.6354/supp-16 Supplemental Information 16 Table S2. Fold change of JcDof genes between five typical flower stages.

10.7717/peerj.6354/supp-17 Supplemental Information 17 Fig. S1. Detailed information of 15 motifs identified in this study.

10.7717/peerj.6354/supp-18 Supplemental Information 18 Fig. S2. Distribution of physic nut, castor bean, *Arabidopsis* and rice Dof genes in groups.

## Additional Information and Declarations

### Data Availability

The following information was supplied regarding data availability: The accession numbers of the gene sequences described in this study are listed in Table S1.
