# Supplementary material for: Genome-wide identification and comparative evolutionary analysis of the Dof transcription factor family in physic nut and castor bean
Source: PeerJ. 2019 Feb 5;7:e6354. doi: 10.7717/peerj.6354 (PMC6368027; doi:10.7717/peerj.6354)
Supplement: Supplemental Information 1 — The gene model for JcDof6.1. [file peerj-07-6354-s001.pdf]

File S1 The gene model for *JcDof6.1* The coding region is marked with uppercase letters, above which are its deduced amino acids (the DOF domain is shown in red). The transcribed untranslated regions, including 5' UTR and 3' UTR sequences, are marked with lowercase letters. The start and stop codons are marked with **bold** letters

1 tatctatcacttttcttgatcatataattaattcatgttgataggaaattttgatttgaa
1 M D R G W K P N V E I S P S C
61 gcaattttgtttgtg**ATG**GATAGAGGATGGAAGCCTAATGTTGAAATATCACCAAGTTGC
16 P R C G S T N T K F C Y Y N N Y S L T Q
121 CCTAGGTGTGGTTCTACAAACACTAAATTTTGTTACTACAACAACTATAGCTTAACACAA
36 P R Y F C K G C R R Y W T K G G S L R N
181 CCGCGATATTTTTGCAAAGGTTGCCGGAGGTATTGGACTAAAGGTGGATCGTTAAGGAAT
56 V P V G G G C R K N R R G K S L R L S S
241 GTTCCCGTGGGCGGAGGTTGCCGGAAGAATAGAAGAGGAAAATCTTTAAGGCTATCTAGT
76 T H D V H V P H S R S L L G N C G G N N
301 ACTCATGATGTACATGTTCCTCATTCAAGAAGCTTATTAGGTAATTGTGGAGGTAATAAT
96 S N N K G V S P P M E S R N N N S M I S
361 TCTAATAATAAAGGGGTTTCTCCTCCAATGGAGTCTCGTAATAATAATTCCATGATATCG
116 E G S Q I D L A L V Y A N F L N P Q S V
421 GAGGGTTCACAGATTGATCTTGCACTTGTTTATGCAAATTTCTTGAATCCACAATCAGTT
136 D H S K I T T T A A P T T I T A A A A A
481 GATCATTCTAAAATTACTACTACTGCTGCTCCGACTACTATAACTGCTGCTGCTGCTGCT
156 T T T G F E M Q E L G S D F D F S G I S
541 ACTACTACGGGATTCGAAATGCAAGAATTGGGTAGTGATTTTGATTTCAGTGGCATTTCA
176 S A N L E L T S L A M E E G S M N E N D
601 AGTGCAAATTTGGAATTAACTAGTCTTGCTATGGAAGAAGGCTCTATGAATGAAAATGAT
196 H Q L M Y Y C G V D S S T N K Q Q L A S
661 CATCAATTGATGTATTATTGTGGAGTGGACTCATCCACAAATAAACAACAACTTGCTAGT
216 N F K I Q E S T S G L P P L P G E E I L
721 AACTTTAAGATTCAAGAATCCACAAGTGGGTTGCCACCATTGCCAGGAGAAGAGATTTTG
236 W D D D N T G N L Q V T Q E P V L G L E
781 TGGGATGATGATAATACTGGTAACTTGCAGGTCACACAAGAGCCAGTTCTTGGACTTGAA
256 N D Q D P N N L L F G D W S P F D L S S
841 AATGATCAAGATCCTAATAATCTATTGTTCGGTGATTGGAGTCCGTTTGATTTGTCTAGC
276 D D T F S R S *
901 GATGATACTTTTTCAAGAAGT**TGA**tatataactaaatgtgatttttacttttacttttat
961 ccctaaattgtatatgttttaaaacgagtactaccacatgagttcacaataatgcatacg
1021 gg
