# Supplementary material for: Genome-wide identification and comparative evolutionary analysis of the Dof transcription factor family in physic nut and castor bean
Source: PeerJ. 2019 Feb 5;7:e6354. doi: 10.7717/peerj.6354 (PMC6368027; doi:10.7717/peerj.6354)
Supplement: Supplemental Information 2 — The gene model for JcDof6.2. [file peerj-07-6354-s002.pdf]

File S2 The gene model for *JcDof6.2* The coding region is marked with uppercase letters, above which are its deduced amino acids (the DOF domain is shown in red). The transcribed untranslated regions, including 5’ UTR, intron and 3’ UTR sequences, are marked with lowercase letters. The start and stop codons are marked with **bold** letters

1 ctttcttgctttctcctttctctctctctctctctctctctggatctttctctgcaaa
61 tactaaaactcctcctcttgttgatttgattctccacttgtgtgtattatctcacaaaaa
121 ttaagaaccactcaatcaacctttacaagaaaaaagaaaagagagaaagctatagctcaa
1 M V F S S I P A Y L D P A N W Q Q
181 gaattgaacc**ATG**GTTTTCTCTTCTATCCCAGCTTATCTTGATCCAGCCAACTGGCAACA
18
241 Agtaaggatttcttgaattttttttggtccttaaggtttctttatttctttgcctaaaaa
301 gcaaaagattgtttcaaaagataaatattcttgttacttttatttcttgtatctttattt
18 Q P N N H H Q
361 tgaatataccagatatttgttcttctttctattttacagCAACCAAATAATCACCACCAG
25 P G G T S G A N T H Q L P P P P P P P P
421 CCTGGCGGAACTAGTGGTGCAAACACTCATCAGCTTCCTCCACCTCCACCTCCACCACCA
45 L P Q P H G S G G A G S I R P G S M A D
481 CTTCCTCAACCTCATGGAAGCGGAGGTGCGGGCTCGATCCGACCTGGTTCAATGGCAGAT
65 R A R L A N I P M P E T A L K C P R C E
541 CGAGCCCGTCTGGCTAACATACCTATGCCAGAGACAGCATTAAAATGCCCAAGATGTGAA
85 S T N T K F C Y F N N Y S L T Q P R H F
601 TCAACAAACACAAAATTTTGCTATTTCAACAACTACAGTCTTACGCAGCCTCGACACTTC
105 C K T C R R Y W T R G G A L R N V P V G
661 TGCAAAACCTGTAGAAGGTACTGGACAAGAGGAGGTGCTCTAAGGAATGTCCCAGTTGGT
125 G G C R R N K R S K G S N S K S P V S G
721 GGTGGTTGTAGGAGAAACAAAAGAAGCAAAGGAAGCAACTCAAAATCTCCGGTCAGTGGT
145 D R P T G S G S S S T V S S N S G T S D
781 GATCGCCCAACGGGTTCTGGTTCCTCAAGTACCGTATCATCTAATAGTGGAACCAGTGAT
165 M L G L V P Q V P P L R F M A P L H H L
841 ATGCTAGGCCTTGTACCACAGGTTCCACCGTTAAGGTTCATGGCTCCTCTTCATCATCTA
185 S E F A T G D I G L N Y G A L S G Q V G
901 TCCGAATTTGCTACTGGAGATATCGGGTTAAACTATGGTGCCCTTTCAGGTCAAGTTGGA
205 G P S D L N F Q I G S A L A S V G L G G
961 GGACCAAGTGACTTGAATTTTCAAATAGGGAGTGCTTTAGCTAGTGTTGGTCTTGGTGGT
225 S G G G C G S G S G S G V G G S L L S V
1021 AGTGGTGGTGGTTGTGGAAGTGGCAGTGGCAGTGGCGTTGGTGGTTCACTTTTGTCAGTG
245 G G L E Q W R L H Q G P Q F P F L G G L
1081 GGTGGTCTAGAGCAGTGGAGGTTACATCAAGGGCCGCAATTTCCTTTCTTGGGTGGGTTA
265 D S S S S N S G L Y P F E S S G G G E P
1141 GATTCTTCTTCTTCTAATTCAGGGTTATACCCATTTGAAAGTAGTGGTGGTGGTGAACCA
285 S G Y G G G V G Q V R P K I S S S L T T
1201 TCTGGTTATGGTGGCGGGGTAGGTCAAGTCAGGCCAAAGATATCAAGTTCTTTAACAACC
305 Q F A S V K M E D N P E L N L S R Q L L
1261 CAGTTCGCATCAGTGAAAATGGAAGATAATCCAGAATTGAATTTGTCAAGACAGCTTCTG
325 G M T G N D H Y W T G T A W T D L S S F
1321 GGTATGACAGGGAATGATCACTATTGGACTGGAACTGCATGGACAGATCTTTCTAGTTTT
345 S S S S T S N P L *
1381 AGCTCTTCTTCTACTAGCAATCCATTA**TAA**gtcttttgccatggaatgaagctcatgaag
1441 ctaagttttttttttttttttttttcctctcctttttctgaagtaggatctcactgttt

1501 ctcagcatcgatcttcttgtaattccatatctcaaagaacttatagttcttgtgtttaag
1561 ttatatggaagagctcagaagatgaatcaagaaatttgcttctctaaagacatatgagct
1621 agaaaaccctagaattatagcttttgcattttatt
