# Supplementary material for: Genome-wide identification and comparative evolutionary analysis of the Dof transcription factor family in physic nut and castor bean
Source: PeerJ. 2019 Feb 5;7:e6354. doi: 10.7717/peerj.6354 (PMC6368027; doi:10.7717/peerj.6354)
Supplement: Supplemental Information 3 — The gene model for JcDof7.2. [file peerj-07-6354-s003.pdf]

File S3 The gene model for *JcDof7.2* The coding region is marked with uppercase letters, above which are its deduced amino acids (the DOF domain is shown in red). The transcribed untranslated regions, including 5' UTR, intron and 3' UTR sequences, are marked with lowercase letters. The start and stop codons are marked with **bold** letters

1 atatccctcagaactcgtttcagtgtcctccagattctctctctctccataaaaggcc
61 catctctgctcttttgccacccacacaaacacaaacacaaacaatccatacccaattcca
121 cattcttctttgctgtccattttctcaactcttttaatctttatcttcttctttctcgaa
181 tattgatcataactaaccaaaagcaaagcagaacaaaacaaagaagaacgaaacctaaat
1 M P S D S S S T E T R R L
241 tacccacttcccatttcccaaa**ATG**CCATCAGACTCTTCTTCCACTGAAACTAGAAGATT
14 T K P H N T G A P P P D Q E H L P C P R
301 AACTAAACCCCATAACACAGGAGCTCCACCGCCAGACCAAGAACACCTTCCATGCCCGCG
34 C D S T N T K F C Y Y N N Y N F S Q P R
361 CTGCGATTCTACTAACACTAAGTTCTGCTATTACAACAACTATAATTTTTCCCAGCCTCG
54 H F C K S C R R Y C P P G G T L R A I P
421 TCATTTCTGTAAGTCTTGTCGCCGTTACTGTCCCCCCGGCGGCACCCTTCGTGCCATTCC
74 V G G G T R K N A K R S R T T S S G C T
481 GGTTGGTGGTGGCACTCGGAAAAATGCTAAAAGATCACGCACCACTTCTAGTGGTTGTAC
94 V V G P M T A N T A D H N I P L P A T P
541 TGTAGTAGGGCCTATGACGGCCAACACAGCTGACCATAACATTCCATTACCGGCTACACC
114 V L V P L M A K Q G N S I Q F G C G G G
601 AGTACTGGTCCCACTTATGGCCAAACAAGGAAATTCTATACAGTTTGGCTGCGGTGGCGG
134 D G K G N G C G S S G N S T V S G S F T
661 TGATGGGAAGGGGAATGGGTGTGGTTCTAGTGGTAATTCTACAGTATCTGGTAGCTTTAC
154 S L L N T Q G P G F L A L S G F G L G L
721 TTCTTTGTTGAATACTCAGGGCCCTGGATTTCTAGCATTGAGTGGGTTTGGGCTTGGACT
174 G S G F E D M G F G L A R G V W P F P G
781 TGGATCTGGGTTTGAAGATATGGGCTTTGGGCTTGCAAGAGGAGTCTGGCCTTTTCCCGG
194 V G D G G A G G V G S N G G S A G G M S
841 TGTAGGAGATGGTGGTGCTGGCGGTGTTGGTAGCAATGGCGGTTCTGCTGGAGGAATGAG
214 N T W Q F E S G D N G F V G G D C F S W
901 TAACACGTGGCAATTTGAGAGTGGTGATAATGGATTTGTAGGTGGGGATTGCTTTTCTTG
234 P D L A I S T P G N G L K *
961 GCCAGATCTTGCTATTTCAACCCCAGGAAATGGTCTTAAA**TGA**gatattgttcttttagt
1021 agttcttgtttttctctgatgggtttagacccaattctaggaaaaaaaaaaaaaaaat
1081 taaagaaccttgtttgatgggtttagttaattaattactacttactattaagtgttgaga
1141 agataattaggtttgaaattagaaagaaaaatatgtaacttttctttactctatttcatg
1201 tgattttgttagtttgatgtacctctttttgtgtagttttcatatacatgccacccttta
1261 aattctcttttcctctctagcaaatttttaatacaaactttttattgaatgattatggac
1321 cgactaatcttaatttaatatgaaattcacattttcttctattcatttcatgaatgggta
1381 caaatggaatttccctttttagagtttgcagcatatacttcaatttgtatttaatatataa
1441 taattgactgatttgatttttgaggcccacatttaaattttaattcttagtataatttat
1501 aatgatttgctagttcaacattaattgagtgcaatcattctcattgcataag
