# Supplementary material for: Genome-wide identification and comparative evolutionary analysis of the Dof transcription factor family in physic nut and castor bean
Source: PeerJ. 2019 Feb 5;7:e6354. doi: 10.7717/peerj.6354 (PMC6368027; doi:10.7717/peerj.6354)
Supplement: Supplemental Information 4 — The gene model for JcDof7.3. [file peerj-07-6354-s004.pdf]

**File S4** The gene model for *JcDof7.3* The coding region is marked with uppercase letters, above which are its deduced amino acids (the DOF domain is shown in red). The transcribed untranslated regions, including 5' UTR, intron and 3' UTR sequences, are marked with lowercase letters. The start and stop codons are marked with **bold** letters

1 ttttttttttttttttgcaaacttaattaactgaatttaactacttacaaactatcattt<br>
61 ctctgtgcaggagctcaaactattggtgttaacttatggcagagttgctgacaaccttcc<br>
121 ccttcctcatcaagtaatactgtgtgcaaatccaagaacattcaaagaattgaaaaacga<br>
1 M P S D S S S T A T<br>
181 aacctaaattacccacttcccatttcccaaa**ATG**CCATCAGATTCTTCTTCCACGGCAAC<br>
11 R R L T K P H N T G A P P A D Q E H L P<br>
241 TAGAAGATTAACCAAACCCCATAACACAGGAGCTCCACCGGCAGACCAGGAACACCTTCC<br>
31 C P R C D S T N T K F C Y Y N N Y I F S<br>
301 ATGCCCGCGCTGCGATTCTACTAACACTAAGTTCTGCTATTACAACAACTATATTTTTC<br>
51 Q P R H F C K S C R R S W T H G G T L R<br>
361 CCAGCCTCGTCATTTCTGTAAGTCTTGTCGCCGTTCCTGGACCCACGGCGGCACCCTTCG<br>
71 D I P V G G D T R K N A K R S R T S S S<br>
421 TGACATTCCGGTTGGTGGTGACACTCGGAAAAATGCTAAAAGATCACGCACCAGTTCTAG<br>
91 G F T V V G P M T A T T G N H N L P L P<br>
481 TGGTTTTACTGTAGTAGGTCCTATGACGGCCACCACAGGTAACCATAACCTTCCATTACC<br>
111 A T P L L A P L M A N Q A S S I Q F G C<br>
541 GGCTACACCACTACTGGCCCCACTTATGGCCAATCAAGCATCGTCTATACAGTTTGGCTG<br>
131 G G G D G K G N V S G S S G N S T V S G<br>
601 CGGTGGCGGTGATGGGAAGGGTAATGTGAGTGGTTCTAGTGGTAATTCTACAGTATCGGG<br>
151 S F T S L L N T Q G P G F L A L S G F G<br>
661 TAGCTTTACTTCTTTGTTGAATACTCAGGGCCCTGGGTTTCTAGCATTGAGTGGGTTTGG<br>
171 L G L G S A F E Y M G F G L A R G V W P<br>
721 GCTTGGACTTGGATCTGCGTTTGAATATATGGGCTTTGGGCTTGCAAGAGGAGTCTGGCC<br>
191 F P G V G D G G A G G V G G N G G S A G<br>
781 TTTTCCCGGTGTAGGAGATGGTGGTGCTGGTGGTGTTGGTGGCAATGGCGGTTCTGCTGG<br>
211 G M S N T W Q F P F Y S L Q Y I F Q F V<br>
841 AGGAATGAGTAACACGTGGCAATTTCCCTTTTATAGTTTGCAGTATATATTTCAATTTGT<br>
231 F N I *<br>
901 ATTTAATATA**TAA**taattgactgatttgatttttgaggcccacatttaaattttaattcc<br>
961 tggcaaaatttataatgatttgctagttcaacattaattgagtgcaatcattctcatagc<br>
1021 ata
