# Supplementary material for: Genome-wide identification and comparative evolutionary analysis of the Dof transcription factor family in physic nut and castor bean
Source: PeerJ. 2019 Feb 5;7:e6354. doi: 10.7717/peerj.6354 (PMC6368027; doi:10.7717/peerj.6354)
Supplement: Supplemental Information 5 — The gene model for JcDof7.4. [file peerj-07-6354-s005.pdf]

File S5 The gene model for *JcDof7.4* The coding region is marked with uppercase letters, above which are its deduced amino acids (the DOF domain is shown in red). The transcribed untranslated regions, including 5' UTR, intron and 3' UTR sequences, are marked with lowercase letters. The start and stop codons are marked with **bold** letters

1 cttatggttatggttcactctatctttagccttttagcagcatttttgcttagtattgag
61 ctccatgactttgcaggtatgatatattatttatttatttattttttgcaaacttaatta
121 actgaatttaactacttataaactatcatttctctgtgcaggagctcaaactagtggtgt
181 taacttatggcagagttgctgacaaccttccctttcctcatcaagtaatactgtgtgcaa
241 atccaagaacattcaaagaattgaaaaacgaaacctaaattacccacttcccatttccca
1 M P S D S S S T A T R R L T K P H N T G
301 aa**ATG**CCATCAGATTCTTCTTCCACGGCAACTAGAAGATTAACCAAACCCCATAACACAG
21 A P P A D Q E H L P C P R C D S T T T K
361 GAGCTCCACCGGCAGACCAGGAACACCTTCCATGCCCGCGCTGCGATTCTACAACAACTA
41 F C Y Y N S Y N F S Q P R H F C K S C R
421 AGTTCTGCTATTACAACAGCTATAATTTTTCCCAGCCTCGTCATTTCTGTAAGTCTTGTC
61 R Y W T H G G T L R D I P V G G G T R K
481 GCCGTTACTGGACCCACGGCGGCACCCTTCGTGACATTCCGGTTGGTGGTGGCACTCGGA
81 N A K R S R T T S S G C T V V G P M T A
541 AAAATGCTAAAAGATCACGCACCACTTCTAGTGGTTGTACTGTAGTAGGTCCTATGACGG
101 N T G D H N L P L P A T P V L V P L M T
601 CCAACACAGGTGACCATAACCTTCCATTACCGGCTACACCAGTACTGGTCCCACTTATGA
121 N Q A T S I Q F G C G G G D G K G N V C
661 CCAATCAAGCAACGTCTATACAGTTTGGCTGCGGTGGCGGTGATGGGAAGGGTAATGTGT
141 G S S G N S T V S G S F T S L L N T Q G
721 GTGGTTCTAGTGGTAATTCTACAGTATCTGGTAGCTTTACTTCTCTGTTGAATACTCAGG
161 P G F L A L S G F G V G L G S G F E D M
781 GTCCTGGATTTCTAGCATTGAGTGGGTTTGGGGTTGGACTTGGATCTGGGTTTGAAGATA
181 G F G L A R G V W P F P G V G E G G A G
841 TGGGCTTTGGGCTTGCAAGAGGAGTCTGGCCTTTTCCCGGTGTAGGAGAAGGTGGTGCCG
201 G V G S N G G S A G G M S N T W Q F E S
901 GTGGTGTTGGTAGCAATGGCGGTTCTGCTGGAGGAATGAGTAACACGTGGCAATTTGAGA
221 G D N G F V G G D C F S W P D L A I S T
961 GTGGTGATAATGGATTTGTAGGTGGGGATTGCTTTTCTTGGCCAGATCTTGCTATTTCAA
241 P G N G L K *
1021 CCCCAGGAAATGGTCTTAAA**TGA**gatattgttcttttagtagttcttgtttttttctctta
1081 taggtttagacttagacccaattctaggaaaaaaaaaaaaaaaaaaaaaattaaagaacc
1141 ttgtttgacgggtttagttaattaattactacttactattaagtgttgagaagataatta
1201 ggt
