# Supplementary material for: Genome-wide identification and comparative evolutionary analysis of the Dof transcription factor family in physic nut and castor bean
Source: PeerJ. 2019 Feb 5;7:e6354. doi: 10.7717/peerj.6354 (PMC6368027; doi:10.7717/peerj.6354)
Supplement: Supplemental Information 6 — The gene model for RcDof02. [file peerj-07-6354-s006.pdf]

File S6 The gene model for *RcDof02* The coding region is marked with uppercase letters, above which are its deduced amino acids (the DOF domain is shown in red). The transcribed untranslated regions, including 5' UTR, intron and 3' UTR sequences, are marked with lowercase letters. The start and stop codons are marked with **bold** letters

```
   1 tccctctctctctctccctctcttctattgcctccttgatccatcagaaacaccacaaca
   1                                                         M  S
  61 caagtttaaggataccatctcatcatcacattaactttattacaagtagcagaaATGTCC
   3 E  G  I  K  L  F  G  K  T  I  P  L  P  A  L  Y  H  L  S  S
 121 GAAGGGATTAAGCTGTTTGGCAAGACCATTCCATTGCCAGCTCTTTACCACTTATCTTCT
  23 A  N  D  P  C  S  Q  A  T  G  D  D  L  D  D  F  S  D  K  T
 181 GCTAATGACCCTTGTTCACAAGCAACAGGAGATGATCTCGATGACTTCTCCGATAAAACT
  43 T  A  S  L  H  E  Y  D  S  V  K  E  Q  Q  A  Q  D  T  N  R
 241 ACTGCTTCTTTACATGAATATGACTCTGTAAAGGAACAACAAGCCCAAGATACGAATAGG
 301 gtatgttttcgtgtatgtttatggatataagaatgattgagcttgaattcctcgttggtc
 361 atatgtgttttagtatttgcaaattctaagaacaattcatctctctttttctaaatatca
 421 tggaagtttcttgtctttcagattttggtagccaattttatattgttaggttattttttt
 481 atcaactcattcctagtttttctgttgtttctggtggctggaatgtctatacgttgacct
 541 caagctaattggcttccattttttaaataaactacatttcctttccggtataggaactggg
 601 tgagctgtatcttcactattgctacccttccaagttcatttcgtattaggtaattgctgc
 661 atagaattctttgaagatctccagctaccagacttaataagagatcttgctgtatcccac
 721 tgtccaggcgatatttcatactaaagagctcattatgaatttagttattttgagttcagg
 781 aaatttcgcaaggactggtgagaaggacatttgatctttttattatttagaaaaaagaac
 841 atttcagtatttcagaattgctttctgaatagagtcttcacattatttgacaagtgatcc
 901 agcaaattaagacttcaattttacagaatattgataatttgtggcctataacttcttgta
 961 tttcttttaggatcagaaaagacaagatttcgtaactttcgatagttactgagataggtt
1021 ttggtgttttcttatgtttgtttgaactaccaatttttccagcttggttacttgtcttct
  63                  E  P  S  G  K  E  V  T  D  D  K  Q  E  V  S
1081 ttgatctttgctatagGAACCTTCAGGAAAAGAAGTCACTGATGATAAACAAGAAGTTAG
  78  T  S  N  Q  E  A  E  D  L  K  D  P  T  T  T  Q  S  R  I  N
1141 CACCTCAAATCAAGAAGCTGAGGACTTAAAAGATCCAACAACAACACAATCACGAATTAA
  98  E  K  P  E  T  S  S  A  N  R  E  T  S  P  S  K  E  S  S  E
1201 TGAAAAGCCTGAAACTTCCTCAGCGAACAGAGAAACTTCACCATCCAAAGAAAGCTCTGA
 118  N  G  E  Q  Q  N  E  T  S  N  N  S  Q  E  K  T  L  K  K  P
1261 GAATGGAGAACAACAAAATGAGACAAGTAATAACTCGCAAGAAAAGACCTTAAAGAAACC
 138  D  K  I  L  P  C  P  R  C  N  S  M  D  T  K  F  C  Y  Y  N
1321 TGACAAAATACTCCCATGCCCTCGATGTAATAGCATGGATACAAAATTCTGCTATTATAA
 158  N  Y  N  V  N  Q  P  R  H  F  C  K  N  C  Q  R  Y  W  T  A
1381 CAATTATAATGTCAATCAGCCCCGCCATTTCTGCAAGAACTGTCAAAGATACTGGACTGC
 178  G  G  T  M  R  N  V  P  V  G  A  G  R  R  K  N  K  S  S  S
1441 TGGAGGAACCATGAGGAATGTACCTGTTGGCGCTGGACGGCGAAAAAACAAGAGCTCTTC
 198  A  S  H  Y  R  H  I  I  V  S  E  A  L  R  T  A  Q  G  H  A
1501 CGCATCACATTACCGGCACATAATTGTATCAGAAGCTTTACGAACAGCACAAGGCCATGC
 218  L  N  G  V  H  N  P  S  F  G  S  S  S  T  V  L  T  F  G  A
1561 TTTGAATGGGGTTCACAACCCTTCTTTTGGAAGTAGCAGTACTGTCCTTACCTTTGGTGC
 238  D  S  P  L  C  E  S  V  A  S  V  L  N  L  S  E  K  N  G  Y
1621 AGATTCTCCTCTTTGCGAATCTGTAGCTTCCGTGCTGAATCTTTCAGAGAAAAATGGATA
 258  H  R  P  E  Q  R  I  L  V  P  C  G  R  T  A  D  N  G  D  D
1681 CCATAGACCTGAACAAAGAATTCTTGTTCCTTGTGGAAGAACAGCTGATAACGGGGACGA
 278  R  L  S  R  N  S  V  T  A  S  N  A  L  E  K  G  G  K  R  V
```

1741 TAGATTGAGTAGAAATTCTGTCACAGCTTCGAATGCATTAGAGAAGGGAGGTAAAAGGGT
298 S Q E T V T K N Y Q G I A P Q I P C F P
1801 TTCGCAAGAAACAGTTACTAAAAATTATCAAGGCATTGCTCCTCAGATACCATGCTTTCC
318 G P P W P Y P W S S P L P T P P T F C P
1861 AGGGCCACCTTGGCCTTATCCATGGAGTTCCCCATTGCCTACTCCACCTACTTTTGCCC
338 S G F P V S F Y P A P A Y W G C T V P N
1921 TTCAGGATTTCCAGTATCATTTTATCCTGCACCAGCCTATTGGGGTTGTACTGTTCCAAA
358 P W N V P P C L S P Q S P S V N H R A A
1981 TCCATGGAATGTTCCTCCATGTCTATCTCCACAATCGCCTTCAGTAAATCACCGCGCAGC
378 A S L S P T S P L G K H S R E G N I L K
2041 GGCGAGCTTGAGTCCTACGTCTCCATTAGGGAAACATTCAAGGGAAGGTAATATCCTGAA
398 P V N S E E P S G E S N C T E R S V L A
2101 ACCTGTCAATTCCGAAGAGCCTTCGGGAGAGAGCAATTGCACGGAAAGAAGTGTTTTGGC
418 P K A L R I D D P T E A V T S S I W A T
2161 TCCAAAAGCTTTGAGGATTGATGACCCAACCGAAGCTGTAACAAGCTCAATATGGGCAAC
438 L G I K C E K T N L N G G L F E G F Q S
2221 TCTTGGGATCAAGTGTGAAAAAACAAATTTGAATGGAGGACTCTTTGAAGGATTTCAGTC
458 K N E N R N Y I A E R K S S V L Q V N P
2281 GAAAAATGAAAACAGAAATTACATAGCTGAGAGAAAATCTTCGGTGTTGCAAGTCAATCC
478 A A L S W S L N F H E S T *
2341 GGCTGCCTTGTCGTGGTCTCTCAACTTCCATGAGAGCACC**TGA**tgaaaggaattgagatg
2401 catgtacactgttgcctctcacacaacaacacaatgtttttcccgaacaagcaacgaatg
2461 gaccgacttgtatcagttgtggagtatattccaatatagaggaaaacccaaaaaaatagg
2521 aacctatcttaccttacaggagaccatagcctctaatcgccttcatagcataatgatcaa
2581 ccggtgttttgccttgaccttagatggtttttttctttcaagtttttgtgtacatatata
2641 tgtgagagtgtgtgacatgtaccttgaaacaaaagagaatcttggatgtcataaaattca
2701 aaaataactggaaaatttcagttcaataacttttatggctctaggc
