# Supplementary material for: Genome-wide identification and comparative evolutionary analysis of the Dof transcription factor family in physic nut and castor bean
Source: PeerJ. 2019 Feb 5;7:e6354. doi: 10.7717/peerj.6354 (PMC6368027; doi:10.7717/peerj.6354)
Supplement: Supplemental Information 7 — The gene model for RcDof05. [file peerj-07-6354-s007.pdf]

File S7 The gene model for *RcDof05* The coding region is marked with uppercase letters, above which are its deduced amino acids (the DOF domain is shown in red). The transcribed untranslated regions, including 5' UTR, intron and 3' UTR sequences, are marked with lowercase letters. The start and stop codons are marked with **bold** letters

1 aacaattctttaattcctttgcactttttagagcttgattgattgcatatgtatgtatat
61 ataagcacattctcttcccttctcctttctgcagccctttcctttttctttcaagctcttc
1 M A N N Q E E G I K L F G T T I T
121 tctttcttta**ATG**GCTAATAATCAGGAAGAGGGTATTAAGCTCTTTGGAACGACAATTAC
18 L H G K Q E V K E D Q N K E N S T P E K
181 GTTGCATGGTAAGCAAGAAGTTAAAGAAGACCAAAACAAAGAAAATTCAACGCCTGAAAA
38 I I P C P R C K S M E T K F C Y F N N Y
241 GATTATACCATGCCCCAGATGCAAGAGCATGGAGACTAAATTTTGTTACTTTAACAACTA
58 N V N Q P R H F C K G C Q R Y W T A G G
301 TAATGTTAATCAGCCTAGACACTTCTGCAAGGGCTGCCAAAGATACTGGACGGCAGGTGG
78 A L R N V P V G A G R R K T K L P G R P
361 GGCTCTTCGTAATGTTCCTGTTGGTGCCGGTCGCCGTAAAACCAAGCTACCTGGACGCCC
98 D G C L Y D G S S G V D P F E L D G R V
421 AGATGGATGCTTGTATGATGGCTCTAGTGGAGTGGATCCATTTGAGCTTGATGGCAGGGT
118 I V E E W Q V A S H G G F R H V F P V K
481 GATCGTTGAGGAATGGCAGGTTGCATCCCATGGTGGTTTCCGGCATGTTTTCCCCGTGAA
138 R R R S N T V
541 GCGGAGGCGGAGCAACACAGgttgtgcaatgtactgattttgacatcttgtctttttcct
601 ttcaaccatattttagagaatatatgcaatatagacagatatataaaactaccgcacgtc
661 tagcttattagtcttgttttcgcagtacataaatataataaacttgcagaagatgacaat
721 tcttgttaatcctggttttcatttttctttttctgtttaatgttcgtagtgtacagtttcg
781 acgacatataagtgcatacatattttatgcgtagagcacatccagaatcaattatttaga
145 Q H E A Q
841 tgcttcataacatgtcttagggatggtgtgtttcccatattgcagTTCAACATGAAGCTC
150 *
901 AG**TGA**ttacgatcatattttggtgttgagagatgtatattttaatggctgaccataaaat
961 atgagtaggtgatccagatcaatctcatcatcatcgagaggctatgatccaattcaatca
1021 aatttgatgctttggagagtcacaagatcgatcagatgcgggcatgcagttgatcttgat
1081 cagtgtcgatcgagctgattg
