# Supplementary material for: Genome-wide identification and comparative evolutionary analysis of the Dof transcription factor family in physic nut and castor bean
Source: PeerJ. 2019 Feb 5;7:e6354. doi: 10.7717/peerj.6354 (PMC6368027; doi:10.7717/peerj.6354)
Supplement: Supplemental Information 8 — The gene model for RcDof06. [file peerj-07-6354-s008.pdf]

File S8 The gene model for *RcDof06* The coding region is marked with uppercase letters, above which are its deduced amino acids (the DOF domain is shown in red). The transcribed untranslated regions, including 5' UTR, intron and 3' UTR sequences, are marked with lowercase letters. The start and stop codons are marked with **bold** letters

1 atcggttcttatatttaaacagccattaacaggttattatatctacacagcattataata
61 tctaacagattagtcttttggtttcacataataggtgtctttagtgggattggagctaga
121 attaataataagggaaacaaaaaaagagaaactgtttaccgtgtaaatacatgtatgtac
181 actgcatgcaggtcaacagaaacagtaaaaccaattggaaacacatgtactgaatcatct
241 ctgcctcctcagtaccaaaaatacgccactgacctacaagccacgcgtctagccgtcatt
301 aaacattgtattaccaccgcctgtgggtgtgcaggagaccaaagagcaaacgcacttcct
361 ttaaaacccatctctctatttccattcgcattcctccaaaatcattactcgcttcctctc
1 M P T
421 tttgcctcctccaaattccttctcttcttcgaaaagacacccaaaatcgaa**ATG**CCAACT
4 E S T P G Q N N T N P V S H S H P P P K
481 GAATCAACTCCAGGCCAAAACAACACCAACCCAGTCTCACACTCGCACCCACCGCCAAAA
24 L A E P L P C P R C D S N N T K F C Y Y
541 CTGGCTGAGCCACTCCCGTGCCCAAGGTGTGATTCTAACAACACAAAATTTGCTACTAC
44 N N Y N L S Q P R H F C K A C R R Y W T
601 AATAACTACAACCTTTCTCAGCCACGCCATTTCTGCAAGGCATGTAGACGTTACTGGACC
64 H G G T L R N V P I G G G T R K N S K R
661 CATGGTGGCACTCTTCGTAACGTACCTATTGGCGGTGGAACCCGCAAGAACTCGAAACGT
84 S R S Y S S S I T T S T T T T S A S A L
721 TCTCGCTCTTATTCTTCTTCGATCACCACCTCCACCACTACTACTTCAGCTTCTGCTTTG
104 S S L N T P D Q P D Q S L P V L A I P E
781 TCATCTTTGAATACTCCAGATCAGCCCGATCAATCTTTGCCTGTCCTTGCTATTCCTGAG
124 S V L T A K S E N L S D N W N L N D E K
841 TCTGTTTTGACAGCCAAAAGTGAGAACTTGTCAGATAATTGGAATCTAAACGATGAGAAA
144 V N L V S Q N G N F I S L L S S Q Q G Q
901 GTTAATCTTGTGAGCCAAAACGGAAACTTTATCTCGCTGTTGAGCTCTCAGCAGGGACAA
164 G F M G M V G Y G P G F G Y G F C D T G
961 GGGTTCATGGGGATGGTTGGTTACGGGCCAGGTTTTGGATATGGGTTTTGTGATACGGGA
184 R E N W V Y P G M A Y V N G G D A V E D
1021 AGAGAGAATTGGGTTTATCCAGGCATGGCCTATGTTAATGGAGGTGATGCTGTAGAGGAT
204 G T S S G C N T W Q Q V E V G D A G G G
1081 GGTACTTCTTCAGGGTGCAACACATGGCAGCAAGTGGAGGTGGGTGATGCTGGTGGTGGG
224 L V D G E N N C L S W P G L A I S T P G
1141 TTAGTTGATGGAGAAAATAACTGCCTTTCTTGGCCTGGTCTTGCAATTTCCACACCAGGA
244 K G F K *
1201 AAAGGTTTCAAG**TGA**agaattggcttttagccattttaaaatttttttgttttaaaaaggg
1261 gctttcttgtaacgttttctttctctttttttgga
