# Supplementary material for: Genome-wide identification and comparative evolutionary analysis of the Dof transcription factor family in physic nut and castor bean
Source: PeerJ. 2019 Feb 5;7:e6354. doi: 10.7717/peerj.6354 (PMC6368027; doi:10.7717/peerj.6354)
Supplement: Supplemental Information 9 — The gene model for RcDof13. [file peerj-07-6354-s009.pdf]

File S9 The gene model for *RcDof13* The coding region is marked with uppercase letters, above which are its deduced amino acids (the DOF domain is shown in red). The transcribed untranslated regions, including 5' UTR, intron and 3' UTR sequences, are marked with lowercase letters. The start and stop codons are marked with **bold** letters

```
   1 tccaacacccacccctacttaccctgcattacacacatggtctcttgcgatcaactctgc
  61 ttgatctcttctcttcttatatatatttgcatacttaaaacataatgcgtgtttacaaaa
   1  M  F  T  S  D  H  H  Q  I  M  L  Q  C  S  A  R  P  L  P  M
 121 tATGTTCACTAGTGACCACCACCAAATCATGTTGCAGTGTTCTGCTAGGCCTTTGCCAAT
  21  E  G  K  W  K  T  H  V  E  L  A  P  S  C  P  R  C  A  S  S
 181 GGAGGGAAAGTGGAAAACCCATGTTGAACTTGCTCCTAGTTGCCCCCGTTGTGCCTCTTC
  41  N  T  K  F  C  Y  Y  N  N  Y  S  L  S  Q  P  R  Y  F  C  K
 241 CAATACTAAATTCTGTTATTACAACAACTACAGCTTGTCTCAGCCTAGATATTTTTGTAA
  61  G  C  R  R  Y  W  T  K  G  G  S  L  R  N  V  P  V  G  G  G
 301 AGGCTGCAGACGGTATTGGACTAAAGGCGGTTCTCTAAGAAATGTTCCTGTTGGAGGTGG
  81  C  R  K  S  R  R  A  K  S  S  R  G  A  V  S  M  N  Y  G  K
 361 CTGTCGTAAAAGTCGCAGAGCTAAGTCTTCTCGTGGCGCAGTTTCGATGAATTATGGCAA
 101  N  F  N  G  P  L  S  G  S  S  C  S  S  N  M  D  S  T  V  Q
 421 GAATTTTAATGGCCCGTTGAGTGGTAGTTCTTGTTCGTCTAACATGGACTCTACGGTTCA
 121  E  T  G  S  N  N  D  G  S  E  I  D  L  A  V  V  F  A  K  F
 481 AGAGACTGGAAGTAATAATGATGGTTCGGAAATTGATTTGGCCGTCGTTTTCGCTAAGTT
 141  L  N  Q  E  S  S  F  Q  P  E  F  V  G  K  E  L  P  D  E  P
 541 CTTGAATCAAGAATCGAGTTTTCAACCTGAGTTTGTTGGGAAAGAATTGCCTGATGAGCC
 161  S  D  Q  E  L  V  S  V  A  N  S  L  S  P  P  D  S  D  A  V
 601 TAGTGATCAAGAACTGGTAAGTGTGGCCAATTCTTTAAGTCCTCCTGACAGTGATGCAGT
 181  I  E  C  Q  N  L  I  G  E  S  I  Q  E  S  N  D  L  L  Q  A
 661 CATCGAATGCCAGAACCTGATCGGCGAAAGCATCCAAGAATCTAATGATTTACTACAAGC
 201  L  L  V  A  G  N  Q  D  Q  E  Q  G  D  Q  E  M  I  M  E  S
 721 GTTGCTTGTTGCTGGAAATCAGGATCAGGAACAAGGAGATCAAGAAATGATCATGGAAAG
 221  Q  D  M  N  E  F  G  L  Q  T  L  L  G  D  E  I  V  Q  D  A
 781 TCAAGACATGAATGAATTCGGATTACAAACTTTGCTAGGCGATGAAATAGTGCAGGATGC
 241  L  W  S  E  A  E  T  S  L  S  N  V  T  W  Q  I  P  V  D  Q
 841 TTTGTGGTCTGAAGCTGAAACATCTCTGTCGAATGTTACATGGCAAATACCAGTTGATCA
 261  V  Q  P  Q  G  F  D  S  F  S  V  D  D  Q  L  K  I  P  T  N
 901 GGTACAGCCGCAAGGATTTGATTCATTCTCAGTGGATGATCAACTTAAGATTCCAACGAA
 281  L  I  N  D  N  A  W  S  S  F  D  L  S  A  G  F  E  V  F  S
 961 TCTTATAAATGATAATGCTTGGAGTTCTTTCGATCTCTCTGCAGGATTTGAGGTTTTCTC
 301  R  S  Y  *
1021 GAGATCTTATTAGacgtataattcacaggaacatttactttcaatttttcttctataatt
1081 tacatatttttgctctctcttcaaagaaatttgatttttactgtcacgtttcccatgaaa
1141 acagtcatggtatatagatactgcagatagagatagtgcaataaaacgatatatacaaat
1201 attccccctgtcattcatcagtttggttgtatctatggatcaaagcaataagattaacaa
1261 atatcacttattatatta
```
