# Supplementary material for: Genome-wide identification and comparative evolutionary analysis of the Dof transcription factor family in physic nut and castor bean
Source: PeerJ. 2019 Feb 5;7:e6354. doi: 10.7717/peerj.6354 (PMC6368027; doi:10.7717/peerj.6354)
Supplement: Supplemental Information 10 — The gene model for RcDof17. [file peerj-07-6354-s010.pdf]

File S10 The gene model for *RcDof17* The coding region is marked with uppercase letters, above which are its deduced amino acids (the DOF domain is shown in red). The transcribed untranslated regions, including 5’ UTR, intron and 3’ UTR sequences, are marked with lowercase letters. The start and stop codons are marked with **bold** letters.

```
   1 cagctctctctctctctatctatctccttgtagactcacttgagtagtgactttttcttg
  61 ccttcttattcacaacccacgccactccatttcctacatgacaaattcaatctccttcat
 121 tcacttctgtccctctttacaatcctcttcttctgtcaccatagaattccagtttccctc
 181 ctcttactacttgtctccatcatcttcttctattcttgcatggtcagtgaagctgccaat
 241 atgaaataaatttaagtgtggtgtgaagtgttgctgaattggttttgctcgagtctgttg
   1                                           M  S  L  T  S  L
 301 tttccttttgtcctttttgttgctagaacttgttttggtttaATGAGTCTTACTTCACTT
   7 Q  V  C  M  D  S  T  D  W  L  Q
 361 CAAGTTTGCATGGATTCCACTGATTGGCTTCAGgtaaagctatatctcattacttacttg
 421 agcttgattattggacatgatgtgttaggttatattgaatatgtacctgtaaaagtacaa
 481 atggtgtaaataatgtaatggggacaattttgttttatttccttgactgttcttgataag
 541 gcatatgtaagtaaaggcttaaactggcatgaaattgtatctgaactatatgttctcaag
 601 aacaagttatcatgaattgaaatgaactgcagcaccgaaaattgttgaaatgaaacgata
 661 ttcttcttttatatccatgaaattcttgctgcacaagtatgataagatatcctataatta
 721 aattcgaccttctggtttttcataaatttggaaaccttatgcacgtacattttgaaagt
 781 ttcttgttcttggcatgtgtgaatctctggcattttggaattaattaaattcagacatt
 841 ctagctatctagatgtgtgaaagactgaaagaatgtctctggtccttttgcattattaat
 901 atctttaaaaggacaaaggaatttcacaattttcaattctctaaaacccttaaattattg
 961 caattgtatgctgataggaacatgtctgacacatttctccatcataattttcttttcttt
  18                         G  T  I  H  E  E  T  G  M  D  S  S
1021 cttttcttgctttccttgtgtcagGGTACGATTCACGAGGAGACTGGAATGGATTCTTCT
  30 S  P  S  G  D  M  L  T  C  S  R  P  L  I  E  R  R  L  R  P
1081 TCCCCTTCGGGGGATATGCTAACATGTTCAAGGCCATTGATAGAGAGGAGGCTTAGGCCA
  50 P  H  D  Q  A  L  K  C  P  R  C  D  S  T  H  T  K  F  C  Y
1141 CCGCATGACCAAGCTCTAAAGTGTCCAAGGTGCGACTCAACACACACCAAATTTTGTTAC
  70 Y  N  N  Y  S  L  S  Q  P  R  Y  F  C  K  T  C  R  R  Y  W
1201 TACAACAATTACAGTCTTTCTCAGCCAAGGTACTTCTGCAAGACTTGCAGAAGGTACTGG
  90 T  K  G  G  T  L  R  N  I  P  V  G  G  G  C  R  K  N  K  K
1261 ACTAAAGGGGGGACTTTAAGGAACATTCCTGTTGGTGGTGGGTGTAGAAAGAATAAGAAA
 110 V  A  K  K  S  N  S  N  E  H  H  Q  P  I  N  Q  N  N  L  G
1321 GTAGCTAAAAAATCGAACAGTAATGAACATCATCAACCCATTAACCAGAACAACCTCGGA
 130 S  S  S  S  H  H  N  P  T  D  L  H  L  S  F  P  D  Q  V  Q
1381 TCATCATCTTCTCATCATAATCCTACTGATCTTCACCTTTCATTTCCTGATCAAGTCCAA
 150 F  S  H  L  N  N  I  L  N  T  Q  G  T  L  A  N  P  T  F  M
1441 TTCTCACATCTTAATAACATACTTAACACTCAAGGGACACTTGCAAACCCTACTTTCATG
 170 E  S  K  Y  S  I  G  M  L  E  N  P  R  P  I  D  F  M  E  S
1501 GAGAGTAAGTACAGCATCGGTATGCTTGAAAACCCTAGGCCTATTGACTTTATGGAGAGC
 190 K  L  E  A  I  V  G  S  S  S  T  S  R  N  Y  D  F  M  G  N
1561 AAGTTAGAAGCAATAGTTGGATCATCAAGCACTTCTAGGAACTATGATTTTATGGGGAAT
 210 S  D  M  G  M  V  S  G  L  G  D  Q  I  N  H  H  H  H  H  H
1621 AGTGATATGGGCATGGTTAGTGGGCTTGGAGATCAAATTAATCATCATCATCATCATCAT
 230 H  H  H  Q  G  L  A  P  N  Y  H  G  L  C  S  P  F  G  M  S
1681 CATCATCACCAGGGATTAGCACCAAACTATCACGGTCTTTGCTCTCCATTTGGTATGTCC
 250 M  D  G  S  N  G  T  F  M  E  T  C  Q  R  L  M  L  P  Y  D
1741 ATGGATGGGAGTAACGGAACGTTCATGGAAACTTGCCAAAGGTTAATGCTTCCGTATGAC
```

270 H Q G N D E Q N T I D V K P N A K L L S
1801 CATCAAGGGAATGATGAGCAAAACACAATCGATGTCAAGCCCAATGCGAAGCTCTTATCC
290 L E W Q E Q G Q G C S H D G G K D T F G
1861 CTTGAATGGCAAGAGCAAGGTCAAGGGTGCTCTCATGATGGTGGGAAAGACACATTTGGG
310 Y L N N L G S S W T G M M N G Y G S S T
1921 TACTTAAACAATTTGGGATCATCATGGACCGGGATGATGAATGGTTATGGATCATCAACA
330 T N P L V *
1981 ACCAACCCTTTGGTG**TAA**tctaaaagaacatcaattcgcctttgataatggaaaggtttt
2041 gttattttctcttttggtgcttctttctattataaggtggtcatatatatgaaattatca
2101 ttgacttgacaaaggagcagcagcagaaggggtgtgtgaatgagaagtttaaagagtcca
2161 aaaatgactgcctttagtatcttactgttt
