# Supplementary material for: Genome-wide identification and comparative evolutionary analysis of the Dof transcription factor family in physic nut and castor bean
Source: PeerJ. 2019 Feb 5;7:e6354. doi: 10.7717/peerj.6354 (PMC6368027; doi:10.7717/peerj.6354)
Supplement: Supplemental Information 11 — The gene model for RcDof18. [file peerj-07-6354-s011.pdf]

File S11 The gene model for *RcDof18* The coding region is marked with uppercase letters, above which are its deduced amino acids (the DOF domain is shown in red). The transcribed untranslated regions, including 5’ UTR, intron and 3’ UTR sequences, are marked with lowercase letters. The start and stop codons are marked with **bold** letters

1 tgggtgggtggctggctggacgatgatgatgatggcgatggtgataaaaaagaatccgtt
61 gcagattttatagaaacccaaagaaaggaaagtactcgaattttctcttaataactgcaa
121 atattttatttttatatttatatgataatttattattttcattttaattcgctttctct
181 gcgtgcaagctttatatataactaacatctgatcatctctccttactccaatttcatcat
241 tgccactctctctctctctcttttccatatttctttctttcaagactattatactttgc
1 M L S F F L
301 tgctttatatatttaaagagctgcccttcttctctcactttgc**ATG**CTCTCTTTCTTTCT
7 L S L N L S F L N Y T N R L S F K K D T
361 ACTTTCTTTGAATCTCTCTTTTCTTAACTACACCAATAGATTGAGTTTCAAGAAAGACAC
27 V Y R P K T T R K E R K G L I W F L A W
421 TGTTTATCGCCCAAAAACAACAAGGAAAGAAAGAAAGGGTTTGATTTGGTTTCTTGCATG
47 M D P S S G Q H Q
481 GATGGATCCTTCTAGCGGACAACACCAGgtagggttattcacagcatggatatatatata
541 tatatatatatatatatatgtttccactataatcctttcttctttgtttctgctatctaa
601 ttaacatacctctggattttcttttcttttctttctttctttcaagtgattttgatgagt
661 tcttttggcctcctctcttttttcccttttttattttggaaggctctccttcttttagatc
721 atttggtctctcatttacaacgatttttttctttttttcttaacctttttttttttgtgtg
781 ttcattaattccatggggatagcttggattgatgaatgaatcacatgaatttgattttca
841 cttggaaaagaaagtacagttgcttaatgatataggttttcaatcatttctgagtctaat
901 acccatcatttctctccttgatctacactaaacaattctctttatgatttttctttttctt
961 gaagtcatgtatggagaggtgaaatttataacaaagaaaaaagataaaaattccttctgc
1021 tatatttctattttgcttgtcaagaaaaatgagagaaagctaaaaacaaacatgtaatta
1081 atcctctttttagtccctagcttttgttcttgttctaggatagtaaataggtatcataag
1141 gtatatataatactttttccctttttatcaatttttctgataactatttaaacaattcaaga
1201 ttaagaggaacacaattagggtcttaaaaggatgtgattgatttcttgcaattacttgta
1261 tttctatcaaatgaatatgtttgaagtacatatacactttgttaggtaagagataaaagt
1321 catttgatgcaatgtggtctaatcgttacatctctttctgtttgttgtttctttctttat
56 E I P T H S L E N M L V C S K
1381 ttttaaccaattcagGAAATTCCTACACATTCATTGGAGAATATGTTGGTTTGCTCAAAA
71 P H Q E R K P R P Q P E Q A L K C P R C
1441 CCACATCAAGAAAGAAAACCAAGACCACAACCAGAACAAGCTCTAAAATGTCCAAGATGT
91 D S T N T K F C Y Y N N Y S L S Q P R Y
1501 GATTCTACCAACACTAAATTCTGCTACTACAACAATTACAGCCTTTCTCAGCCAAGGTAT
111 F C K S C R R Y W T K G G T L R N V P V
1561 TTTTGCAAGTCATGCAGAAGGTACTGGACTAAAGGAGGAACCCTGAGAAATGTTCCAGTA
131 G G G C R K N K R S T K K A Q D H H Q L
1621 GGTGGAGGTTGTAGAAAGAACAAAAGATCAACAAAGAAGGCACAAGATCATCATCAATTG
151 N P H T N P L T G L P P L S Y D S N D L
1681 AATCCTCATACCAACCCACTTACTGGTCTCCCCCCTTTAAGTTATGATTCCAATGACCTT
171 T L A F A R L Q K Q S S G Q L G F D D N
1741 ACTCTTGCCTTTGCTAGACTTCAGAAGCAGTCTAGTGGGCAGTTAGGGTTTGATGACAAT
191 D F S I L G N P S N A H C D I M L G N P
1801 GATTTCTCTATTTTGGGAAACCCCAGCAATGCCCACTGTGATATAATGCTTGGGAACCCT
211 N S I N T S A S T P S F L D A L R S G F
1861 AACAGCATTAACACTTCAGCTTCAACTCCATCATTTCTTGATGCTTTAAGGAGTGGGTTT

231 L D T Q N N N F Q N L Y Y G Y G N G S I
1921 CTTGATACCCAAAATAACAACTTTCAGAATTTGTATTATGGGTATGGAAATGGGAGCATT
251 D D V E N S G G V C V S G E M M L P Y D
1981 GATGATGTGGAAAATAGTGGAGGGGTTTGTGTTAGTGGAGAAATGATGTTGCCTTATGAT
271 H Q D V S T S A A T Q A V T V T T M K Q
2041 CATCAAGATGTAAGTACTAGTGCAGCCACACAAGCTGTGACAGTGACAACAATGAAGCAA
291 E F C N G R E D H N S N N S K V L F G F
2101 GAGTTTTGCAATGGTAGAGAAGATCATAACAGCAATAATAGTAAGGTCTTGTTTGGCTTT
311 P W Q I N G N G I S D L D S G R E S W N
2161 CCCTGGCAGATAAATGGTAATGGGATCAGTGATCTTGATTCAGGAAGAGAAAGCTGGAAC
331 N H G L G N S T W H G L I N S P L M *
2221 AATCATGGACTTGGTAATTCAACTTGGCATGGACTTATCAACAGTCCTCTAATG**TAG**tag
2281 aactgcagcagcttaattagctaagaaagaaaaaagaaaaaagaaagaagaaaaagaaag
2341 aagaacctgcaaaaccccaagaacaagaatagagttttcttttcttattattagactcaa
2401 aatctcagtttttaggctcttgagttttgtgttttagaagatttgattccactgaatcat
2461 ctctatcttaaatttttctttttttttttttcttttttttccctctcttgtttcctttta
2521 ttgttttttttcttttatataaatatactaatgaaaatttgtttgctaaccaa
