# Supplementary material for: Genome-wide identification and comparative evolutionary analysis of the Dof transcription factor family in physic nut and castor bean
Source: PeerJ. 2019 Feb 5;7:e6354. doi: 10.7717/peerj.6354 (PMC6368027; doi:10.7717/peerj.6354)
Supplement: Supplemental Information 12 — The gene model for RcDof19. [file peerj-07-6354-s012.pdf]

File S12 The gene model for *RcDof19* The coding region is marked with uppercase letters, above which are its deduced amino acids (the DOF domain is shown in red). The transcribed untranslated regions, including 5’ UTR, intron and 3’ UTR sequences, are marked with lowercase letters. The start and stop codons are marked with **bold** letters

1 cattctttcttgcactaaagagcagctgcagacaaaggaggtgaattcttccatctctct
61 ttatctatctctctctctctttctctttctgaagtcatcactttcttttctttcttgctc
121 tttctgaatttatggatgctgctccatggccacaggtgggtagtttttctgttcttcttt
181 tttgaaaaaaaaaaagaatttgggggctagctggtgtatttttctatgtagaattgtgaa
241 ttttattgtttaaagctagcttttagggttaactaacataacttggatcatgttctccac
301 tagttaattaattagttttaagagattgcaagttattgctttttgtttcacttttaactt
361 gttctttttttttttttagagatttgttcatgtggaaaggtgtatttccccccaccttgtat
421 ctgagctttgttattagaaggataaaagtttaattcttttgttcttgaaacatgaaagca
481 ctgcacttttgcttttcttatcctttttgctttgtgtgagaactgataaagggattggaa
541 aaggtataatttgttattgtcatttgttcttttgtttttctttttaagaaactcttttc
601 tttcttgtttcttatatttcctttttctaaagttctgctaaagagaagcttggttttat
661 attgtttgatctgtttttttgttgttgttgtttgtctcttgattaaaaccttgtttaatt
721 aaatttaagatttgagattttcgtgttcctgtagcatggatttcgtttcattgtctctct
1 M E E I
781 atttattactagctttagggtttgcaggatattaaactggttaagtcc**ATG**GAAGAAATA
5 S S N A C S R P V Q E R K S R P Q E Q L
841 TCATCTAATGCATGCTCAAGGCCAGTACAAGAGAGAAAATCAAGGCCTCAGGAGCAATTG
25 N C P R C N S T N T K F C Y Y N N Y S L
901 AACTGTCCAAGATGCAATTCAACCAATACCAAATTTTGTTACTACAACAACTACAGTCTA
45 T Q P R Y F C K T C R R Y W T E G G S L
961 ACTCAACCAAGATACTTTTGCAAGACTTGCAGAAGGTATTGGACAGAAGGAGGATCTCTT
65 R N V P V G G G S R K N K R S S T S S S
1021 AGAAATGTTCCTGTTGGAGGAGGTTCGAGAAAGAACAAGAGATCATCAACATCATCTTCT
85 S S I V P A S S S A S I S E L P D L N P
1081 TCTTCAATAGTACCAGCATCATCATCAGCCTCAATATCTGAGCTTCCTGATCTAAACCCA
105 P C I S Q F S S Q N P K I H E G Q D L N
1141 CCATGCATTTCACAGTTTTCCTCTCAAAACCCTAAGATTCATGAAGGGCAAGATCTTAAT
125 L A F S A A M P D S Q A
1201 CTGGCTTTCTCTGCAGCTATGCCGGACAGTCAAGgtatatcttattttcttgaggtaccc
1261 agcactgaaaacatgaacaacaacatcaacactcaacataactcttcttcttcttcttct
137 L E L L R T G I
1321 tcttcttcttctacttcttcttcatatacttcagCTTTGGAGCTGCTAAGAACTGGAATT
145 A S R G L N S F I P S P M P D S N T L Y
1381 GCTTCAAGGGGTTTGAATTCTTTCATTCCATCACCGATGCCGGATTCAAACACACTGTAC
165 S S G F P M Q E F K P T L S F A V E G L
1441 TCTTCAGGGTTTCCAATGCAAGAATTCAAGCCAACACTTAGTTTTGCGGTTGAAGGGCTT
185 G L G N R Y G V R E N D G R V F F P L G
1501 GGACTTGGGAATAGATACGGGGTCCGAGAAAACGATGGAAGAGTTTTCTTTCCTTTAGGA
205 E L K N L S S P S H H H E V D Q N K G Q
1561 GAGTTAAAAAATCTTTCAAGCCCAAGTCATCATCACGAAGTTGATCAAAATAAGGGACAA
225 G T S A A A A T T T T S S T G F W N G M
1621 GGGACTAGTGCTGCTGCTGCTACTACTACTACCTCTTCAACTGGATTTTGGAATGGAATG
245 F G G G G G S W *
1681 TTTGGCGGGGGTGGAGGATCGTGG**TAA**ataaatcaaagatcaagaaagacaagaagacca
1741 agaaactacttttgtatcttcttctcttttctcttctattaccttttacttttacatggt

1801 cgtttacgtgttgaaagttgtccaggactaacagatagggattcttctcttctcttctct
1861 ttcattactatttttgagcagttgtaaaaaaattttgaagctcatttgctttggcttcaa
1921 aagggttttcctttacacatttatatataattggtgtgagagtgagagatagaagaagat
1981 gtgaatacaagtctaatgtgggttttctttcatggcagcatagcatggactgtattcata
2041 aattcttctatctgaattagatcaagtacctgcgctcttgttgtatttggtttccacttt
2101 ccagagttgggcctcagtttatatttttgtttattcaatgcgaaactgagatttcatatc
2161 acatcatatcaaacttcgattcagccaatgatattcttt
