# Supplementary material for: Genome-wide identification and comparative evolutionary analysis of the Dof transcription factor family in physic nut and castor bean
Source: PeerJ. 2019 Feb 5;7:e6354. doi: 10.7717/peerj.6354 (PMC6368027; doi:10.7717/peerj.6354)
Supplement: Supplemental Information 13 — The gene model for RcDof23. [file peerj-07-6354-s013.pdf]

File S13 The gene model for *RcDof23* The coding region is marked with uppercase letters, above which are its deduced amino acids (the DOF domain is shown in red). The transcribed untranslated regions, including 5' UTR, intron and 3' UTR sequences, are marked with lowercase letters. The start and stop codons are marked with **bold** letters

```
   1 ttttctttcttgctttctttctcttgtctctgcaaaaccacaaaaaaaaaaaaaaaaaaa
  61 aaaccactccctcttgttgatgtgattctccacttgtattatctcaccctcaaagagaaa
   1                                                          M
 121 caaaaaaaaaaaaaaaacaacccaaacaacctttacaaaaagagaaagatcaagaaccATG
   2 V  F  S  S  L  P  A  Y  L  D  P  A  N  W  Q  Q  R
 181 GTTTTTCTTCTCTTCCAGCTTATCTTGATCCAGCCAACTGGCAGCAACGAgtaagggtt
 241 tcttcaatttctctctctctttttctttggttcttaaatttttctttgcctaaaggga
 301 aaaatttcaaaagataaatattcttgtttacttttgttttcttctatctttattttgaat
  19                                                            Q
 361 ataccagatatttgatttcggcttttatatttatatcttttttttctctctattttacagC
  20   P  N  H  H  H  H  H  H  H  Q  P  G  A  S  T  G  A  N  A  H
 421 AACCAAATCATCATCATCATCATCATCACCAACCTGGCGCAAGTACCGGTGCAAATGCTC
  40   L  I  P  P  T  P  P  P  P  P  P  P  P  P  P  P  P  P  H  G
 481 ACCTTATTCCTCCTACTCCTCCACCTCCTCCTCCTCCTCCTCCACCACCACCACCTCATG
  60   S  G  G  A  G  S  I  R  P  G  S  M  A  D  R  A  R  L  A  N
 541 GAAGTGGTGGTGCTGGCTCGATCCGGCCAGGTTCGATGGCGGATCGAGCTCGCTTGGCTA
  80   I  P  M  P  E  A  A  L  K  C  P  R  C  E  S  T  N  T  K  F
 601 ACATACCTATGCCAGAGGCAGCATTAAAATGTCCAAGATGCGAATCAACAAACACTAAGT
 100   C  Y  F  N  N  Y  S  L  T  Q  P  R  H  F  C  K  T  C  R  R
 661 TTTGCTACTTCAACAACTATAGTCTCACACAGCCTAGGCACTTTTGCAAAACCTGTAGAA
 120   Y  W  T  R  G  G  A  L  R  N  V  P  V  G  G  G  C  R  R  N
 721 GGTACTGGACAAGAGGTGGTGCCCTAAGAAATGTCCCTGTTGGTGGTGGTTGCAGGAGGA
 140   K  R  S  K  G  S  S  S  S  K  S  P  V  S  S  D  R  Q  T  A
 781 ACAAGAGAAGCAAAGGAAGCAGCAGCTCCAAATCTCCTGTCAGTAGTGATCGCCAAACAG
 160   S  G  T  S  S  T  L  S  S  S  G  T  S  D  I  L  G  L  G  P
 841 CCTCCGGTACTTCAAGCACTCTGTCTTCCAGTGGAACCAGTGATATTTTAGGTCTTGGAC
 180   Q  V  P  P  L  R  F  M  A  P  L  H  H  L  N  E  Y  A  P  C
 901 CACAGGTTCCACCTTTGAGATTCATGGCTCCTCTGCATCATCTTAATGAATATGCTCCAT
 200   D  I  G  L  N  Y  G  A  L  S  A  P  V  G  G  T  S  D  L  N
 961 GTGATATTGGGTTAAATTATGGTGCCCTTTCAGCACCTGTTGGAGGAACTAGTGACTTGA
 220   F  Q  I  G  S  A  L  A  S  T  G  L  G  G  G  S  G  V  A  G
1021 ATTTTCAGATAGGGAGTGCTTTAGCTAGTACTGGTCTTGGTGGAGGTAGTGGTGTTGCTG
 240   G  S  L  L  S  M  G  G  L  E  Q  W  R  L  Q  Q  G  Q  Q  F
1081 GTGGTTCTCTTTTGTCAATGGGTGGTTTAGAGCAGTGGAGGTTGCAACAAGGACAGCAAT
 260   P  F  L  G  G  L  D  P  S  S  S  A  G  L  Y  P  F  E  G  G
1141 TTCCATTCTTGGGTGGGTTAGATCCTTCTTCTTCTGCTGGGTTATACCCTTTTGAGGGTG
 280   A  E  P  S  G  Y  G  G  G  I  G  G  Q  V  R  P  R  T  S  T
1201 GAGCTGAGCCATCTGGTTATGGTGGTGGGATTGGAGGCCAGGTTAGGCCAAGGACATCAA
 300   S  L  T  T  Q  F  A  S  V  K  M  E  D  N  H  E  L  N  L  S
1261 CTTCTTTAACTACCCAATTCGCCTCAGTGAAAATGGAAGACAACCATGAGCTTAATTTGT
 320   R  Q  F  L  G  I  N  N  P  G  S  D  Q  Y  W  S  S  T  A  W
1321 CAAGGCAGTTCTTGGGAATTAATAATCCAGGAAGTGATCAGTATTGGAGTAGTACCGCAT
 340   T  D  L  S  S  F  S  S  S  S  T  S  N  P  L  *
1381 GGACAGATCTTTCTAGTTTTAGCTCTTCTTCTACTAGTAATCCATTATAGatattgtctt
1441 tgctggtctgtgcttaaccatgggttcaagcttcagtttttcctatttccctttcctcag
```

1501 ttaaagaaaatcacaagtagttaggatcatcattttcatcaatcactcgtcttcttgaat
1561 ctccatagctggaaattttactgctcaagttcaagatagaggagtttggaagatggatga
1621 aggagtttgcttctttcaagattaactagaaaaccctagaacggtaggcttttgtctttc
1681 ttctttttttctttttttggtttttctggagttacttcgtttctttcttacttcttattgt
1741 agttttaggttgcgatgtagtagcatgtttaccacagatgagcgtatggtgaatctatgc
1801 gagctagtttgtatcacatacatatgctgatgaaaactatatgtttttgagttccatatg
1861 aacttgtatgattccaaaa
