# Supplementary material for: Genome-wide identification and comparative evolutionary analysis of the Dof transcription factor family in physic nut and castor bean
Source: PeerJ. 2019 Feb 5;7:e6354. doi: 10.7717/peerj.6354 (PMC6368027; doi:10.7717/peerj.6354)
Supplement: Supplemental Information 14 — The gene model for RcDof24. [file peerj-07-6354-s014.pdf]

File S14 The gene model for *RcDof24* The coding region is marked with uppercase letters, above which are its deduced amino acids (the DOF domain is shown in red). The transcribed untranslated regions, including 5' UTR, intron and 3' UTR sequences, are marked with lowercase letters. The start and stop codons are marked with **bold** letters

1 atctttccccttccccttctctccctttaattctttctctcctttttctctctttgcttt
61 gctttcttgcctctggtattctcacttagtgtgattctccaccatatttctttcctaaa
121 gacccatcaaactttacaaaagaatagaaaagaaaaggagaaaaaagatcaaaaaagaaa
1 M V F S S V P I Y L D P P
181 aagaagaaaaaagaattaaaacc**ATG**GTTTTCTCTTCAGTTCCAATCTATTTAGATCCTC
14 N W Q Q
241 CCAACTGGCAGCAGgtaagattttctcttcatcgatttccatccacaaacatatagagtt
301 tcttttcttttatctctctccgaaaaagaaaggaaaacaaacaagagcatatatcataaa
361 gattattattcctattccttctgtaatctttttcttgaatatactacatataatttcctc
18 Q P N
421 tctaatgttactaaattcttgaatttttcttatttttttggtcttacagcagCAACCAA
21 Q Q Q G F T N E T P H F P S L P P P P P
481 ATCAACAACAAGGATTTACCAATGAAACTCCCCATTTTCCATCTCTTCCTCCTCCTCCTC
41 Q V G G S G G S S G S G S I R P G S M A
541 CTCAAGTTGGGGGTAGCGGCGGCAGCAGTGGCAGTGGCTCGATTAGACCTGGTTCGATGG
61 D R A R L A K I P Q P E A S L K C P R C
601 CAGATCGAGCCAGGTTAGCAAAGATACCACAGCCAGAAGCATCTTTAAAGTGTCCGAGGT
81 E S T N T K F C Y F N N Y S L S Q P R H
661 GTGAATCAACAAACACCAAGTTTTGCTACTTCAATAATTATAGCCTTTCTCAACCTCGTC
101 F C K T C R R Y W T R G G A L R N V P V
721 ACTTTTGCAAGACTTGCCGGCGATACTGGACTAGAGGCGGTGCTCTTAGAAATGTTCCAG
121 G G G C R R N K K S K S Q S N S K T P V
781 TAGGAGGTGGATGCAGGAGAAACAAGAAAAGCAAAAGCCAAAGTAACTCAAAGACTCCAG
141 S N T N E R R M G S N S S S T S A V P S
841 TTTCTAATACTAATGAAAGGCGAATGGGTTCAAATTCAAGCTCAACAAGTGCAGTTCCTT
161 D I I S H L P P Q S S H Q L P F F S S L
901 CAGATATTATAAGCCATTTGCCTCCACAATCGAGTCATCAGTTACCTTTCTTTTCTTCTT
181 H N L T Q F G V G N I G L N F G G I Q T
961 TGCATAACCTTACTCAATTTGGTGTAGGCAATATTGGGTTAAACTTTGGAGGAATTCAAA
201 S G A N G Q A D M G L F Q I G S N N N S
1021 CAAGTGGTGCTAATGGACAAGCTGACATGGGATTATTTCAAATAGGAAGTAATAATAATT
221 G M T S S A M L S N G G M Q Q F P F F E
1081 CAGGCATGACTAGTAGTGCTATGTTATCAAATGGAGGAATGCAACAATTCCCTTTCTTTG
241 P P P A T G L Y P F H Q S D Q G I E A S
1141 AGCCACCGCCAGCTACTGGGTTATATCCATTTCATCAAAGTGATCAAGGTATAGAAGCAT
261 P S S M L G D N S Q L I P S M T S S S S
1201 CACCATCTTCCATGCTAGGTGATAATAGTCAGCTTATTCCATCAATGACTTCTTCAAGCT
281 R V S Q S P P V K M E D S N Q Q G L N L
1261 CTAGGGTTTCTCAGTCACCTCCAGTGAAGATGGAAGATAGTAACCAACAAGGGTTAAACT
301 P V V S W Y L R D Q *
1321 TACCGGTAGTTTCTTGGTATCTCAGAGATCAA**TAA**tcagcactggagaggaaacagattt
1381 ttcaggtcccaactcttcttccacttagccatctcttataatttacacttgatcgatcgt
1441 ttcctgattgaagaacgttctaagctcaaaactctatccctatagagcatttgctactta
1501 gcgataatttgacgtaaaattaagttgcattgcatatacgcaacgcacgatctactgaaa
1561 acccagcttaacttgccagttccaaccctcaaaaaacggtgacattgcgcgcttgagttc

1621 aatgaggagctgcctctcttgatcatacgatccgagggaaagatggcagcaaaaaatggt
1681 agaattaatgtttactttaaatttgtttcctgctttgtttgtttcagtcgtgtttctggt
1741 ctgtattattttatggcttgtttgagagtttgtgtaacgagagagatgatggtgtttgtt
1801 atacctgacatggtcatgctgaactattatatcaagataacgcatctt
