# Supplementary figures and images for: Genome-wide identification and comparative evolutionary analysis of the Dof transcription factor family in physic nut and castor bean
Source: PeerJ. 2019 Feb 5;7:e6354. doi: 10.7717/peerj.6354 (PMC6368027; doi:10.7717/peerj.6354)

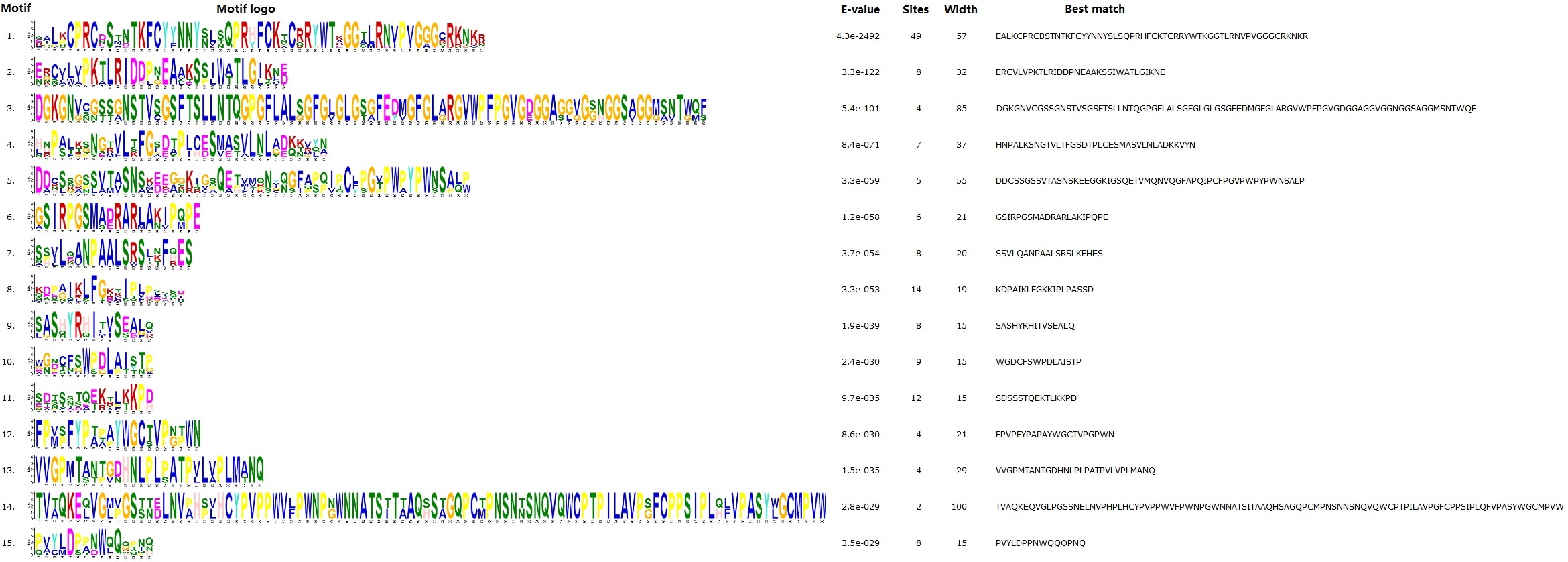

Supplement: Supplemental Information 17 — Detailed information of 15 motifs identified in this study. [file peerj-07-6354-s017.jpg]

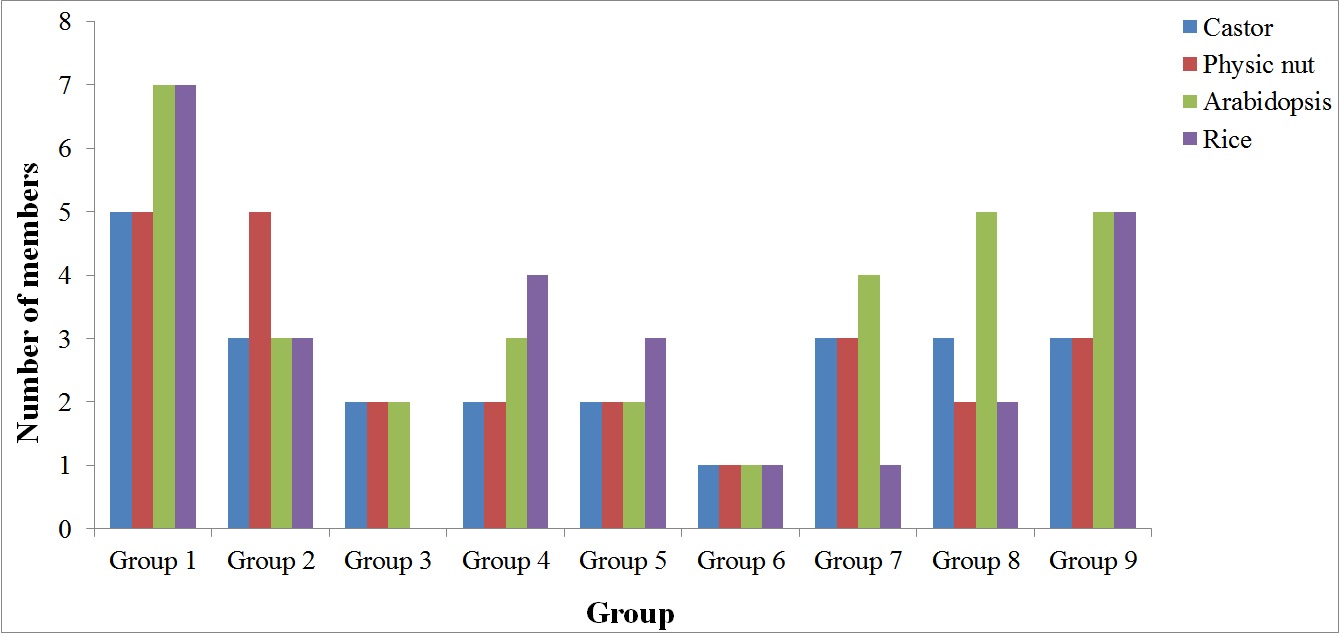

Supplement: Supplemental Information 18 — Distribution of physic nut, castor bean, Arabidopsis and rice Dof genes in groups. [file peerj-07-6354-s018.jpg]
